# Parallel Driving and Modulatory Pathways Link the Prefrontal Cortex and Thalamus

**DOI:** 10.1371/journal.pone.0000848

**Published:** 2007-09-05

**Authors:** Basilis Zikopoulos, Helen Barbas

**Affiliations:** 1 Department of Health Sciences, Boston University, Boston, Massachusetts, United States of America; 2 Program in Neuroscience, Boston University, Boston, Massachusetts, United States of America; 3 New England Primate Research Center, Harvard Medical School, Boston, Massachusetts, United States of America; 4 Department of Anatomy and Neurobiology, Boston University School of Medicine, Boston, Massachusetts, United States of America; James Cook University, Australia

## Abstract

Pathways linking the thalamus and cortex mediate our daily shifts from states of attention to quiet rest, or sleep, yet little is known about their architecture in high-order neural systems associated with cognition, emotion and action. We provide novel evidence for neurochemical and synaptic specificity of two complementary circuits linking one such system, the prefrontal cortex with the ventral anterior thalamic nucleus in primates. One circuit originated from the neurochemical group of parvalbumin-positive thalamic neurons and projected focally through large terminals to the middle cortical layers, resembling ‘drivers’ in sensory pathways. Parvalbumin thalamic neurons, in turn, were innervated by small ‘modulatory’ type cortical terminals, forming asymmetric (presumed excitatory) synapses at thalamic sites enriched with the specialized metabotropic glutamate receptors. A second circuit had a complementary organization: it originated from the neurochemical group of calbindin-positive thalamic neurons and terminated through small ‘modulatory’ terminals over long distances in the superficial prefrontal layers. Calbindin thalamic neurons, in turn, were innervated by prefrontal axons through small and large terminals that formed asymmetric synapses preferentially at sites with ionotropic glutamate receptors, consistent with a driving pathway. The largely parallel thalamo-cortical pathways terminated among distinct and laminar-specific neurochemical classes of inhibitory neurons that differ markedly in inhibitory control. The balance of activation of these parallel circuits that link a high-order association cortex with the thalamus may allow shifts to different states of consciousness, in processes that are disrupted in psychiatric diseases.

## Introduction

Interactions between the thalamus and cortex mediate shifts in our state of consciousness, the sleep-wake cycle, quiet rest, or attention [1]–[3]. Imbalance in the communication between the thalamus and cortex is at the core of a host of psychiatric and neurological conditions [4]–[7]. Yet, most of our ideas about thalamo-cortical communication are based on sensory systems, but little is known about high-order association areas of the cortex that govern our thoughts, emotions, and actions.

Capitalizing on knowledge about the source and type of peripheral signals processed, classic studies in sensory systems have shown that signals from the periphery reach the thalamus and then are relayed to the middle layers of the laminated cerebral cortex [4], [8]–[10]. In turn, a pathway from the cortex emanates from a different layer (VI) and innervates sensory thalamic nuclei. Most sensory and high-order association areas, however, communicate with the thalamus through additional pathways, which engage different cortical layers. In primates, one pathway from the thalamus terminates in the superficial cortical layers [11], and a cortical pathway originates from layer V and projects to the thalamus [8], [12], [13]. In several mammalian species there are, therefore, four pathways that link the thalamus and cortex, two in each direction (e.g., [11], [13]).

The significance of the varied interactions between the thalamus and cortex emerged from functional studies that sorted pathways into ‘drivers’, which ensure efficient passage of signals, and ‘modulators’, which evoke small and graded physiological responses on postsynaptic neurons [14]–[17]. The classical thalamo-cortical pathway that terminates in the middle cortical layers is considered to be a driver pathway, and the reciprocal cortico-thalamic pathway emanating from cortical layer VI is thought to be modulatory [8], [9], [12], [15]. On the other hand, the thalamo-cortical pathway that terminates in the superficial layers is thought to be modulatory, and the cortico-thalamic pathway from layer V appears to be a driving pathway [11], [18]–[21].

The intricate structure and physiology of these pathways underlie complex and dynamic interactions between the thalamus and cortex [22]. Yet a clear link between the structure and function of these pathways is missing. The lack of a holistic understanding of the architecture of these pathways may be attributed to the piecemeal accumulation of evidence from qualitative approaches. Consequently, there is little information on shared or unique pre- and postsynaptic features of these pathways or their interactions with chemically specific neurons and glutamate receptors in the thalamus, which are thought to underlie the dynamic interactions between thalamus and cortex [14]–[17], [23]. These issues have remained largely unexplored in most systems, and are unknown for high-order association cortices, whose interactions with the thalamus play a crucial role in cognitive and emotional processes [24]–[27].

To investigate whether these pathways have similar or distinct structural and neurochemical features, we used as a model system the reciprocal pathways connecting the ventral anterior thalamic nucleus with the prefrontal cortex in rhesus monkeys. The ventral anterior nucleus links prefrontal, premotor and motor cortices and provides an anatomic substrate for the involvement of the prefrontal cortex in cognitive, emotional, and executive processes [25], [26]. This ideal model system made it possible to first study the global architecture of all four pathways of the two circuits that link the ventral anterior nucleus with prefrontal cortex, and then focus on some of their synaptic features. We provide novel evidence for structural, neurochemical and synaptic specificity in two complementary circuits that likely mediate dynamic interactions between the thalamus and prefrontal cortex and may allow recruitment of cortical and thalamic structures in complex behavior.

## Results

### Overview

**Figure 1:**
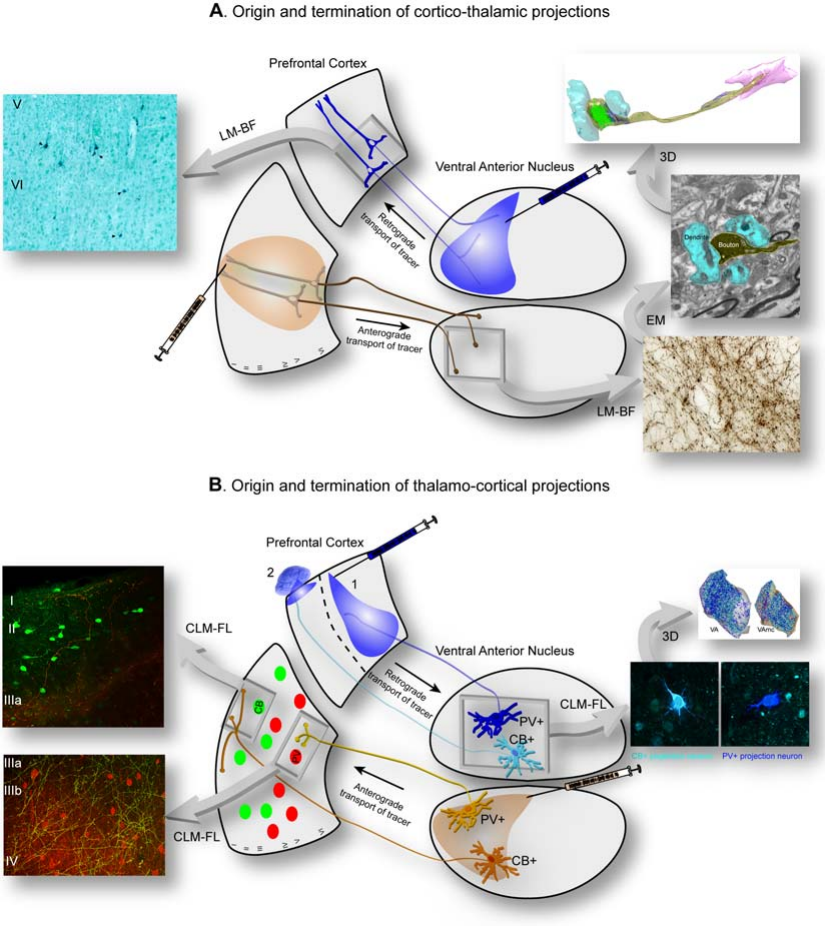
Summary of the experimental approaches for the quantitative study of bidirectional connections of the ventral anterior nucleus with the prefrontal cortex. *A,* *Cortico-thalamic pathways:* Injection of retrograde tracers in the ventral anterior nucleus (blue injection, center-top) labeled projection neurons in prefrontal cortex (left photomicrograph; arrowheads on dark blue neurons), quantitatively mapped under brightfield in the light microscope (LM-BF). Injection of anterograde tracers in prefrontal cortices (brown injection, center-bottom) was employed to determine the quantitative distribution of terminals in the ventral anterior nucleus using brightfield microscopy (LM-BF; right-bottom photomicrograph). Material from the latter cases was used to conduct a detailed analysis of the ultrastructural features of cortical terminals (yellow shading) in the ventral anterior thalamic nucleus and to identify the neurochemical characteristics of their postsynaptic targets (blue shading) in the electron microscope (EM; right-center photomicrograph), and reconstruct synapses in three dimensions (3D; right-top). *B,* *Thalamo-cortical pathways:* To study the origin and laminar termination of thalamo-cortical pathways in the prefrontal cortex we used two approaches. First, injection of retrograde tracers in all layers (blue injection 1, center-top) labeled neurons in the ventral anterior nucleus that project to all layers of prefrontal cortex. Application of the tracer only to the superficial layers of prefrontal cortex, by placing a tracer soaked sponge (∼1mm long) on the surface of the prefrontal cortex (blue injection 2, center-top), labeled projection neurons in the ventral anterior nucleus that project only to the superficial layers. The distribution and density of CB+ (light blue) and PV+ (dark blue) thalamic projection neurons in the ventral anterior nucleus were estimated using fluorescence or confocal laser microscopy (CLM-FL) and three dimensional reconstruction of the nucleus (right, top). In a second approach, injection of anterograde tracers in the ventral anterior nucleus (brown injection, center-bottom) was used to quantitatively map the laminar distribution and measure the size of CB+ (orange) and PV+ (yellow) terminals in the prefrontal cortex, using double labeling immunofluorescence and confocal laser microscopy. The laminar relationship of thalamic axonal terminations in the prefrontal cortex to local inhibitory neurons labeled with CB (green) or PV (red) was studied using fluorescence or confocal laser microscopy (CLM-FL; left photomicrographs).

The ventral anterior nucleus and its medial magnocellular part are situated rostrally in the dorsal thalamus of the rhesus monkey brain. The nuclei had a similar shape across animals, appearing round rostrally, and elongated caudally. Stereological measurements of the nuclei on coronal sections and reconstruction in three dimensions showed that the nuclei were similar in size and shape across animals, with a mean volume of 41±2 mm^3^ (mean±standard error of mean, SEM) for the ventral anterior nucleus, and 24±1 mm^3^ (mean±SEM) for its medial magnocellular part. Since both nuclei established similar patterns of synaptic connectivity with the prefrontal cortex they will be referred together as the ventral anterior nucleus. Figure 1 shows the essence of our experimental approaches to study bidirectional connections of the ventral anterior nucleus with prefrontal cortex and their neurochemical and synaptic features.

### Cortico-thalamic pathways

#### Overview of prefrontal terminations in the ventral anterior nucleus

Labeled prefrontal axons entered the ventral anterior nucleus rostrally and laterally from the adjacent internal capsule passing through the inhibitory thalamic reticular nucleus. A majority of prefrontal cortico-thalamic terminals (≥70%) were found in largely overlapping sites of the rostral half of the nucleus. The topography of prefrontal terminals in the ventral anterior nucleus was analyzed in detail in a previous report [26], and confirmed here. A more detailed account of the quantitative distribution of prefrontal terminals in the ventral anterior nucleus is described in Text S1 and Figure S1.

#### Dual laminar origin and mode of termination of prefrontal neurons projecting to the ventral anterior nucleus

Prefrontal projections originated from pyramidal neurons in layers V and VI and their axons terminated as small and large boutons in the ventral anterior nucleus. The dual laminar origin was shown after injections of retrograde tracers in the ventral anterior thalamic nucleus, which labeled projection neurons in the prefrontal cortex (Fig. 1A). Cortico-thalamic projection neurons were found in both layers V (45%) and VI (55%), in agreement with a previous study [26]. Compared to the cell bodies of projection neurons in layer VI, most layer V corticothalamic projection neurons were larger, in most cases by twofold.

**Figure 2:**
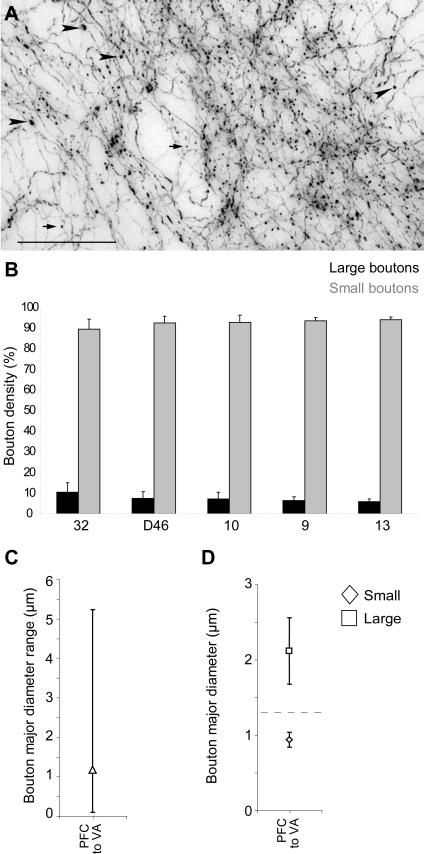
Bouton population analysis of prefrontal terminations in the ventral anterior thalamic nucleus. *A,* The dimorphism of sizes of boutons from prefrontal projections to the ventral anterior nucleus. Brightfield photomicrograph showing BDA-labeled prefrontal axons and boutons from area 32 terminating in the ventral anterior nucleus, as large boutons (black arrowheads), or small boutons (black arrows). Scale bar: 50 µm. *B*, Large and small axonal bouton populations from prefrontal cortices in the ventral anterior nucleus. Normalized density (%) of large and small boutons in the ventral anterior nucleus from prefrontal axons. *C*, Mean major diameter of boutons from axons originating in prefrontal cortices and terminating in the ventral anterior nucleus. Range of diameters of boutons is also indicated (vertical lines). *D*, Cluster analysis of the diameter of boutons (± variance) emanating from prefrontal cortices and terminating in the ventral anterior nucleus (VA).

We measured the size of prefrontal terminals, which in other systems is thought to affect neural dynamics [28]–[30]. Axonal terminals from prefrontal cortex to the ventral anterior nucleus constituted both *en passant* and *terminaux* types and they were polymorphic. Prefrontal terminals fell into at least two distinguishable classes by size, consistent with prefrontal projections to other thalamic nuclei [27], [31]. As shown in Figure 2A, most axonal boutons were small (∼93% of the entire population; mean density: 122,356 boutons/mm^3^; diameter range: 0.10–1.30 µm), but a significant number of large boutons were also present (∼7% of the entire population; mean density: 9,374 boutons/mm^3^; diameter range: 1.31–5.20 µm; Fig. 2B). Scatter and frequency distribution plots showed that the population of boutons was continuous, covering a wide range of sizes (Fig. 2C). However, cluster analysis indicated that there were at least two distinct populations (p<0.00001), one consisting of small boutons (mean diameter±variance = 0.94 µm±0.09), and the other consisting of large boutons (mean diameter±variance = 2.12 µm±0.44; Fig. 2D). Interestingly, although large boutons constituted a small percentage of the total population, they were observed on 35% of the labeled axonal branches. The majority of axons (65%) gave rise exclusively to small boutons, the minority only to large (6%) and the rest (29%) gave rise to a mixture of small and large boutons. Axons containing large boutons were usually thicker than axons with small boutons (diameter of thick axons ≥1.5 µm).

**Figure 3:**
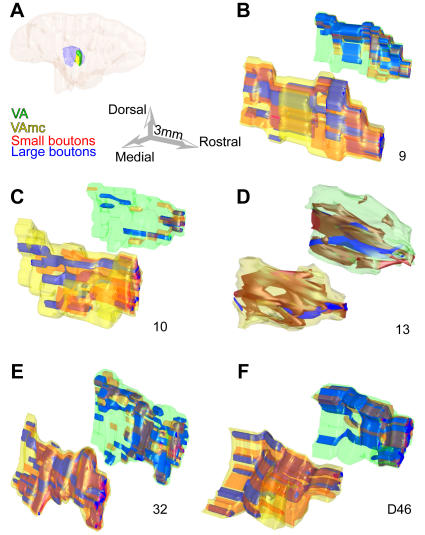
Large and small bouton populations in the ventral anterior nucleus. Separate reconstructions are shown for the principal nucleus (VA) and its magnocellular part (VAmc). *A,* Reconstructed hemisphere of a rhesus monkey brain, shown from the medial surface, which was rendered transparent to show the position of the ventral anterior nuclei (VA: green; VAmc: yellow) surrounded by the thalamic reticular nucleus (purple). *B–F,* Three-dimensional (3D) reconstruction of the topographical distribution of large (blue) and small (red) boutons from prefrontal axons in the ventral anterior nuclei. The VAmc is in the foreground and appears larger in 3D space, but it is actually smaller. For details about the size of each nucleus see text. Numbers below each pair show the prefrontal cortical origin of boutons from areas 9, 10, 13, 32 and D46.

Further comparison of all prefrontal terminations revealed that all labeled bouton populations in the ventral anterior nucleus were similar, regardless of area of origin in the prefrontal cortex, though projections from area 32 contained the highest density of large boutons (Fig. 2B; p = 0.01). In other cortico-thalamic pathways large boutons terminate in aggregated clusters. In contrast, the overall distribution of large terminals from prefrontal cortices was similar to that of small boutons in the ventral anterior nucleus. Large boutons from areas 9, 10, 32 and 46 were more widespread than from area 13 (Fig. 3).

#### Presynaptic features of small and large cortico-thalamic terminals

**Figure 4:**
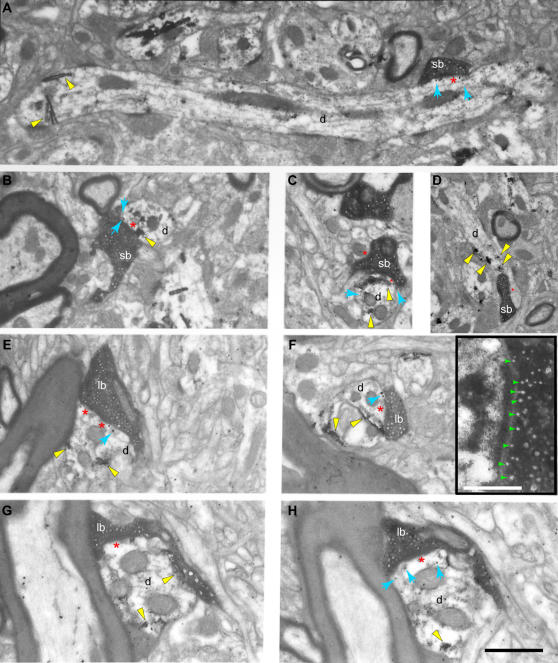
The fine structure of large and small prefrontal terminations in the ventral anterior nucleus. The photographs were obtained from single two-dimensional sections. *A–C*, Electron microscopic (EM) photomicrographs showing small labeled boutons (sb) forming synapses with PV+ dendrites (d; black rods or grains, yellow arrowheads), with metabotropic mGluR1a receptors perisynaptically (black dots, blue arrows). *D,* EM photomicrograph showing a small labeled bouton forming a synapse with a PV+ dendrite (d; yellow arrowheads) without any mGluR1a receptors perisynaptically. *E–H*, EM photomicrographs showing large labeled boutons (lb) forming synapses with CB+ dendrites (d; black rods or grains, yellow arrowheads), with ionotropic NR1 receptors perisynaptically (black dots, blue arrows). The inset in F shows the synapse of that bouton at higher magnification showing docked vesicles (green arrowheads); scale bar: 0.25 µm. G and H show two serial sections of the same large bouton. The TMB labeling of the CB+ dendrite is obvious in G (yellow arrowheads), and the gold labeling of the perisynaptic NR1 receptors is clearly apparent in H (black dots and blue arrows). In many cases labeled receptor molecules were observed presynaptically (e.g., E) or inside axons (e.g., G). Red asterisks (*) indicate postsynaptic densities. Scale bar: 1 µm.

**Figure 5:**
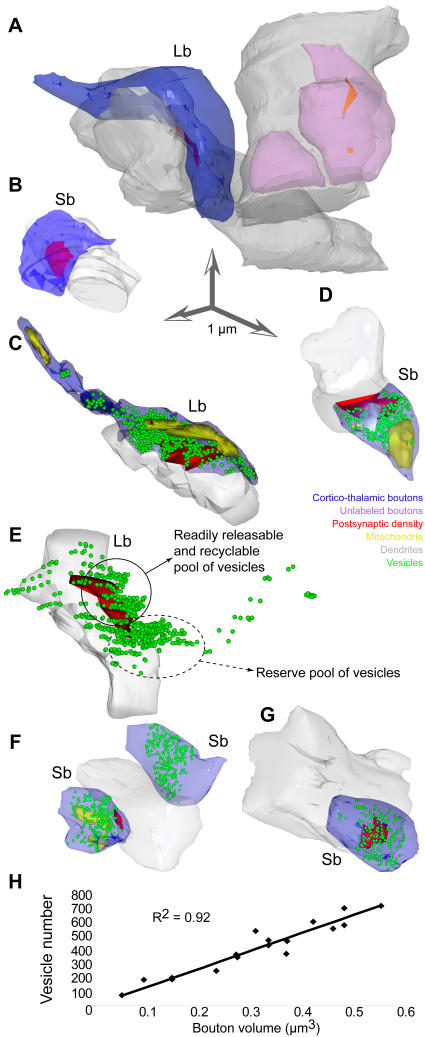
Presynaptic features of large (Lb) and small (Sb) boutons in the ventral anterior nucleus. *A-B*, 3D reconstruction of labeled prefrontal boutons (blue; same as in Fig. 4B, G) forming synapses (red) with PV+ or CB+ dendrites (grey). Unlabeled boutons (pink) were included for comparison. *C–G,* The position of vesicles (green spheres) and mitochondria (yellow structures inside boutons) are shown in the reconstructions. The large bouton in C is a reconstruction of the bouton in Fig. 4E, and the small bouton in D is a reconstruction of the bouton in Fig. 4C. *E,* In most cases with large boutons (Lb), there were two separate clusters of vesicles: a smaller pool (solid circle), adjacent to the postsynaptic density (red), and a larger pool distal to the postsynaptic specialization (dotted circle). E, contains the same structures as C, but is rotated 180^o^ in the y axis, and the bouton and its mitochondria were removed to facilitate distinction of vesicle clusters. Large boutons (Lb) were at least 2 times bigger than small boutons (Sb). *H,* The relationship of bouton volume to vesicular content was linear.

Small and large terminals from prefrontal axons differed significantly in presynaptic features. Evidence was obtained after their three-dimensional reconstruction from serial ultrathin sections photographed in the electron microscope, as summarized in Figure 1A (right panels). Both groups of boutons formed exclusively asymmetric (presumed excitatory) synapses with dendritic shafts of thalamic neurons, but differed in several other aspects. The most numerous group consisted of small boutons, spherical in shape, with round synaptic vesicles and one or no mitochondria (mean volume±SE = 0.15 µm^3^±0.02), and contained on average 306±109 vesicles (1317±115 vesicles/µm^3^; volume, 10.5×10^−6^±0.1 µm^3^/vesicle). Examples of single sections through small boutons are shown in Figure 4A–D, and 3D reconstructions of entire boutons are shown in Figure 5B, D, F, G. The less numerous group consisted of large, round, ovoid, or irregularly shaped boutons, also with round synaptic vesicles, but with at least two or more mitochondria (mean volume±SE = 0.40 µm^3^±0.02), and twice as many vesicles as the small boutons (average 677±65 vesicles; 1368±175 vesicles/µm^3^; volume, 10.9×10^−6^±0.1 µm^3^/vesicle; Fig. 4E–H, Fig. 5A, C, E). There was a positive linear correlation between the number of vesicles and bouton size (Fig. 5H). Comparison of the volumes of labeled boutons with unlabeled boutons forming synapses on the same or neighboring dendrites showed no significant difference between the two populations.

Three-dimensional reconstruction of large boutons revealed, in most cases (∼90% of large boutons), two distinct pools of synaptic vesicles, resembling the readily releasable–recycling pool and the reserve pool [32]–[34], as shown in Figures 4F (inset) and 5E. Such a distinction was not clear in most small boutons even though synaptic vesicles were distributed throughout the terminals forming cluster-like arrangements. Estimation of the distance of a vesicle from the active zone and subsequent cluster analysis suggested separation of vesicles in two distinct groups, with the reserve pool containing vesicles at a distance ∼0.2 µm or further from the active zone. Large boutons formed larger synapses than small boutons (as measured by area of postsynaptic density), but the overall ratio of the synapse to bouton area was constant (0.05±0.01). The vast majority of synapses were macular, forming one identifiable synapse, however, 20% of the large bouton population formed perforated synapses, with two clearly identifiable postsynaptic densities on the same postsynaptic target. Large boutons with large synapses, or two synapses, had more docked vesicles (6–15) compared to small boutons (2–6), in agreement with other studies [35]. Synaptic vesicles were not always associated with synaptic specializations since they were also found in some axons, although their density was higher near the release sites (Fig. 5C–G, green). There was close association of mitochondria with the pool of synaptic vesicles, as seen in Figure 5. In cases of small boutons with no mitochondria the density of synaptic vesicles was lower (p<0.01).

#### Postsynaptic targets of prefrontal axonal boutons onto distinct neurochemical classes of thalamic neurons

Thalamic nuclei in primates have two major neurochemical classes of projection neurons, distinguished by their expression of the calcium binding protein parvalbumin (PV) or calbindin (CB) [18], [19]. In sensory systems, these classes of thalamic neurons correspond to core and diffuse thalamic projection systems. The interaction of these systems with prefrontal pathways is largely unknown.

**Figure 6:**
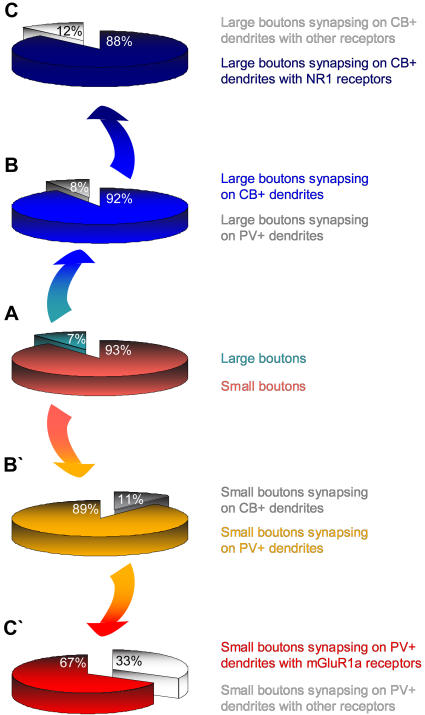
Morphological and neurochemical features of prefrontal cortico-thalamic synapses in the ventral anterior nucleus. *A, center*, Proportion of small (light red) and large (light blue) boutons from prefrontal axons in the ventral anterior nucleus. *B,* Most large boutons formed synapses with CB+ dendrites of thalamic projection neurons (dark blue) and very few established synapses with PV+ dendrites (grey); *C,* most of these CB+ dendrites contained NR1 receptors (navy blue), few had other receptors, but not mGluR1a (light grey). *B̀,* Most small boutons formed synapses with PV+ dendrites of thalamic projection neurons (light orange) and few formed synapses with CB+ dendrites (grey); *C̀,* most of these PV+ dendrites contained mGluR1a receptors (red), and fewer had other receptors (light grey).

We found that large and small boutons from prefrontal axons showed near segregation (∼90%) in their preference for these two neurochemical classes of thalamic neurons, revealed after double or triple labeling (Fig. 4) and synaptic reconstruction. As summarized in Figure 6B, the vast majority of large prefrontal terminals formed synapses with calbindin-positive (CB+) dendritic shafts (∼92%), and the rest (8%) formed synapses with parvalbumin-positive (PV+) dendritic shafts of thalamic neurons. The opposite was true for small prefrontal boutons, where 89% formed synapses with PV+ dendritic shafts and only 11% with CB+ dendrites (Fig. 6B').

Large and small boutons from prefrontal cortices showed further specialization in their postsynaptic targets. As shown in Figure 6C, the vast majority of large boutons (∼88%) formed synapses exclusively with CB+ dendritic shafts containing ionotropic N-methyl-D-aspartate receptors (NMDA-NR1) at the synapse or at sites adjacent to the postsynaptic density (Fig. 4E–H). We did not find large boutons forming synapses with dendritic shafts containing the metabotropic glutamate receptor mGluR1a. In contrast, as seen in Figure 6C', the majority of small boutons (∼67%) established synapses with PV+ dendritic shafts which contained the metabotropic glutamate receptor mGluR1a, located around the postsynaptic specialization (Fig. 4A–C). In this pathway, we found a few small terminals forming synapses with dendritic shafts positive for NR1 receptors. Further details about the size of the postsynaptic targets of prefrontal terminals in the ventral anterior nucleus can be found in Text S2.

In summary, these findings indicate that pathways from prefrontal cortex to the ventral anterior thalamic nucleus are specialized. One pathway preferentially targeted through large terminals CB+ thalamic neurons enriched with ionotropic glutamate receptors at the synapse, consistent with a driving pathway. The other preferentially targeted through small terminals PV+ thalamic neurons with metabotropic glutamate receptors, consistent with a modulatory pathway.

### Thalamo-cortical pathways

#### Neurochemical identity and laminar specificity of thalamo-cortical projections

The thalamo-cortical projections from the ventral anterior nucleus to the prefrontal cortex reciprocated the cortico-thalamic pathways, in agreement with previous studies [25], [26], but their regional density varied. Medial prefrontal areas in the anterior cingulate (areas 24, 32) and the dorsal and medial parts of area 9 included the highest densities of labeled fibers. Orbital regions (areas 13, OPro) and lateral area 46 exhibited moderate projections, and area 10 was sparsely innervated.

**Figure 7:**
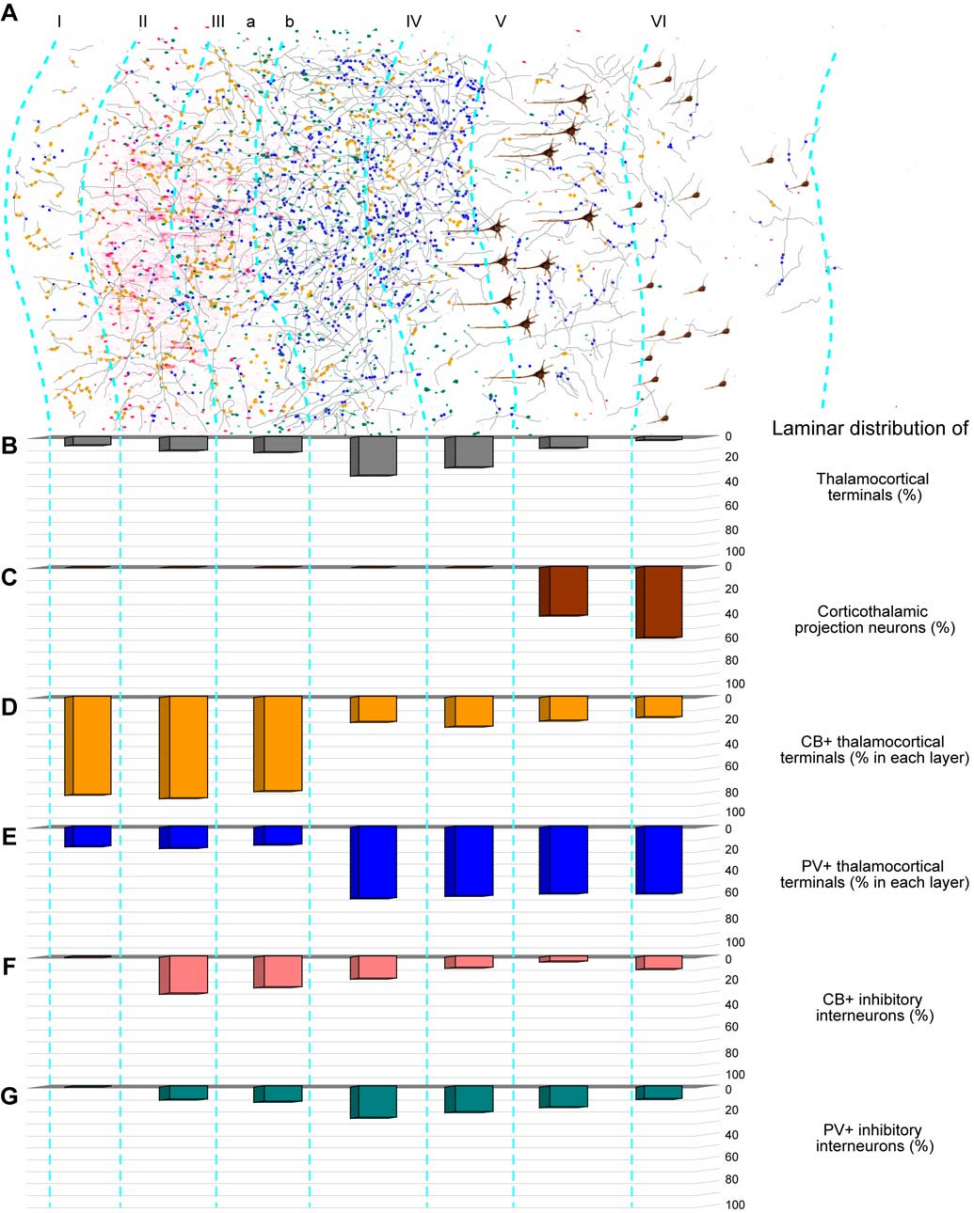
Summary of thalamo-cortical terminals from the ventral anterior nucleus within laminar microenvironments in the prefrontal cortex. *A,* Neurolucida drawing of a strip of prefrontal cortex from area 9, showing the distribution of thalamo-cortical axons (grey), CB+ (yellow dots) or PV+ terminals (blue dots), cortico-thalamic projection neurons (brown), and local inhibitory neurons (red for CB+ and green for PV+). *B–G,* Quantitative laminar distribution (%) of: *B,* thalamo-cortical terminals; *C,* cortico-thalamic projection neurons; *D,* CB+ thalamo-cortical terminals; *E,* PV+ thalamo-cortical terminals; *F,* CB+ inhibitory neurons in prefrontal cortex; *G,* PV+ inhibitory neurons in prefrontal cortex.

Figure 7 summarizes the density and neurochemical features of the pathways from the ventral anterior thalamic nucleus to prefrontal cortex. Thalamo-cortical projections terminated in all layers of the prefrontal cortex (Fig. 7A, B). The majority of thalamo-cortical axons terminated in the middle layers (∼57%; layer, IIIb: 32%; layer IV: 25%). Moderate to high numbers of axonal terminals were found in layers IIIa (13%), II (11%) and V (9%), and in layer I (7%), and the lowest proportion was found in layer VI (3%; Fig. 7A, B), a pattern that was similar throughout the prefrontal cortex.

**Figure 8:**
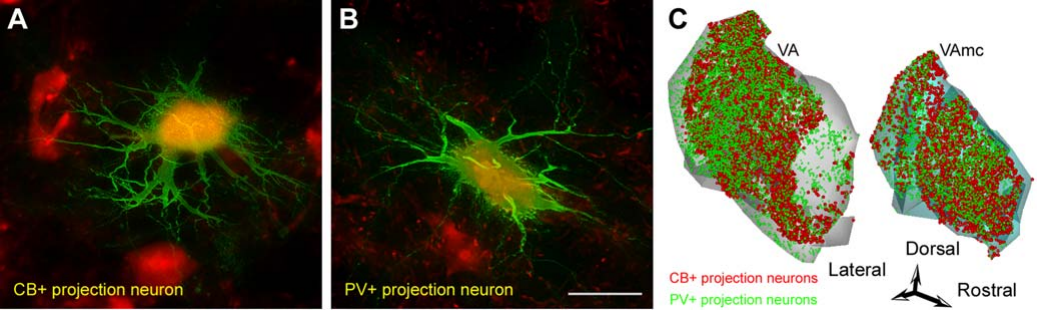
The two neurochemical classes of thalamo-cortical projection neurons in the ventral anterior nuclei labeled with CB or PV. Projection neurons were retrogradely labeled with a tracer (FE; green fluorescence), then immunohistochemically labeled in 300 µm sections for CB or PV (red fluorescence), and then microinjected to show the dendritic tree (LY or Alexa Fluor 488) resulting in yellow fluorescence. *A,* Intracellularly filled CB+ projection neuron. *B,* Intracellularly filled PV+ projection neuron. *C,* 3D-reconstruction of the topographical distribution of the population of CB+ (red) and PV+ (green) projection neurons in the ventral anterior nuclei. The neurons are intermingled in both nuclei. Scale bars: A-B, 50 µm; C, 1.5 mm.

Thalamo-cortical projection neurons positive for PV or CB have a distinct organization in the primate thalamus [18]. Information about their interactions with the cortex has focused on sensory systems, however, their specific interactions with the prefrontal cortex are unknown. We used two approaches to determine the identity of projection neurons in the ventral anterior nucleus belonging to the PV or CB classes, and their axonal terminations in prefrontal cortex, as shown in Figure 1B. First, injections of retrograde tracers in all prefrontal cortical layers labeled retrogradely thalamic projection neurons in the ventral anterior nucleus that projected to all cortical layers. Placement of tracer-soaked sponges on the surface of the prefrontal cortex labeled specifically thalamic neurons projecting to the superficial layers of prefrontal cortex, especially layer I. Stereological analysis revealed that 12% of thalamic projection neurons projected to the superficial layers, though not exclusively, and the other 88% projected to all other layers. The thalamo-cortical projections from the ventral anterior nucleus to prefrontal cortex originated predominantly from the two distinct neurochemical populations of thalamic projection neurons. Evidence was provided after double labeling immunofluorescence to view simultaneously ventral anterior neurons projecting to prefrontal cortex and their label for CB or PV (Fig. 8A, B). The two classes of projection neurons were intermingled in the ventral anterior nucleus (Fig. 8C). These projection neurons were not positive for gamma-aminobutyric acid (GABA), in agreement with thalamo-cortical projections in primates, which are excitatory [18]. Their densities were similar in the ventral anterior nuclei, with CB+ projection neurons being slightly more than the PV+ projection neurons (CB+: ∼51%, 2,125±112 cells/mm^3^; PV+: ∼47%, 1,991±94 cells/mm^3^). A small third population of projection neurons in the ventral anterior nuclei (∼2%) was not labeled by either CB or PV. Quantitative analysis of neurons projecting to the superficial layers of the cortex, which accounted for about 12% of all the projection neurons in the ventral anterior nucleus, revealed that the majority were CB+ (∼60%), a few were PV+ (∼7%), and the rest (∼33%) were not labeled by either antibody.

**Figure 9:**
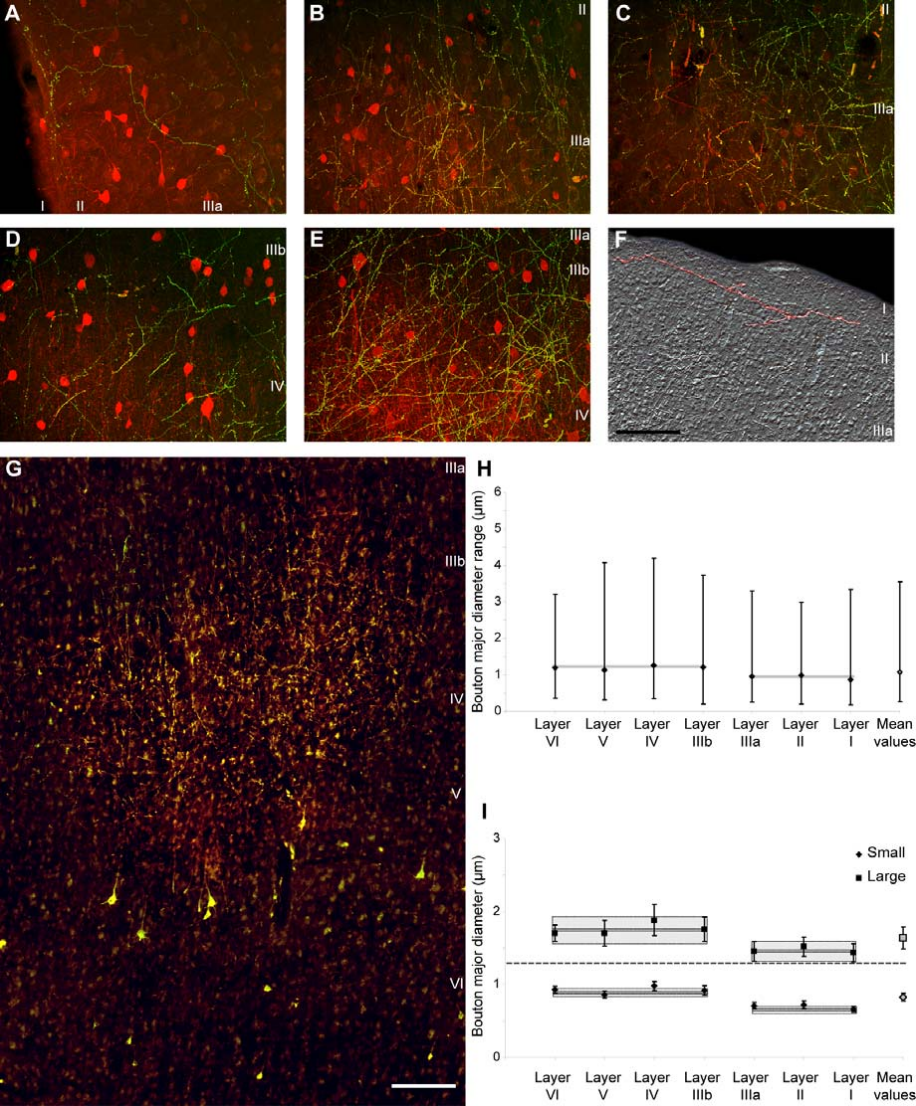
Thalamo-cortical terminations in different layers of the prefrontal cortex. Most thalamo-cortical terminals in the superficial layers are CB+, whereas in the middle-deep layers most are PV+. *A–C*, Thalamo-cortical terminals (green) in prefrontal cortex from the ventral anterior nucleus and their relationship to CB+ processes and local inhibitory neurons (red). Yellow shows double labeled thalamo-cortical terminals that are CB+. Axonal terminations from the ventral anterior nucleus in the superficial layers of: *A,* area 32; *B,* area 9; *C,* area 46. *D–E*, Thalamo-cortical terminals (green) in the prefrontal cortex from the ventral anterior nucleus and their relationship to PV+ processes and local PV+ inhibitory neurons (red). Yellow shows double labeled thalamo-cortical terminals that are PV+. Axonal terminations from the ventral anterior nucleus in the middle-deep layers of: *D,* area 46; *E,* area 9. *F,* Widespread axonal terminations of small and large boutons in the superficial layers of area 9. One thalamo-cortical axon is shown in pseudo-color (red). *G,* Focused axonal terminations in the middle-deep layers of area 9. Labeled cortico-thalamic neurons are seen in layers V and VI. *H,* Mean major diameter of boutons from axons originating in the ventral anterior nucleus and terminating in different layers of the prefrontal cortex. The range of diameters of boutons (vertical solid lines) and the mean values in the superficial and middle-deep layers (grey boxes) are also indicated. *I*, Cluster analysis of the diameter of boutons (± variance) emanating from the ventral anterior nucleus and terminating in different prefrontal cortical layers. Populations of small and large boutons can be identified in all layers; however, boutons in the superficial layers are significantly smaller than in the middle-deep layers. Dark and light grey boxes indicate the mean values±standard error in the superficial and middle-deep layers. Scale bars: *A–F*, 100 µm; *G*, 200 µm.

The above approach provided information on the neurochemical identity of thalamic neurons projecting only to the superficial layers of prefrontal cortex. We then used the reverse approach, namely injected anterograde tracers in the ventral anterior nucleus, and employed double-labeling and confocal laser microscopy to determine the neurochemical identity of thalamic axons positive for CB or PV terminating throughout the layers of prefrontal cortex (Fig. 1B). As summarized in Figure 7D–E, this approach made it possible to quantitatively assess the laminar distribution of all thalamo-cortical terminals. In agreement with the analysis of the thalamic projection neurons, the vast majority of thalamo-cortical boutons terminating in layer I of prefrontal cortices were CB+ (∼82%), fewer were PV+ (16.5%), and only a few (1.5%) were not labeled by either antibody. Interestingly, terminations in all superficial layers (I-IIIa) were similar, as a vast majority of thalamo-cortical boutons labeled with neural tracers were also CB+, and therefore originated from the CB+ population of projection neurons in the ventral anterior nucleus (Fig. 9A–C). In contrast, the majority of thalamo-cortical boutons terminating in the middle and deep layers (IIIb-VI) were PV+ (∼58%), originating from the PV+ population of projection neurons in the ventral anterior nucleus, 21% were CB+, and 21% were not labeled by either antibody (Fig. 9D, E).

It should be noted that this method also labeled the distinct classes of PV+ and CB+ neurons in the cortex, which unlike the thalamic projection neurons, are local inhibitory neurons. However, thalamo-cortical and local inhibitory axons and varicosities were distinguished because the thalamo-cortical axons were double labeled and fluoresced yellow (green fluorescence from the tracer and red fluorescence from CB or PV), whereas the local inhibitory neurons in prefrontal cortex were single labeled for CB or PV (red fluorescence, Alexa Fluor 568; Fig. 9A–E).

#### Structural features of thalamo-cortical terminals in the prefrontal cortex

We measured the size and shape of thalamo-cortical terminals from a bouton population of at least 4,000 labeled profiles per case. Like the cortico-thalamic, the thalamo-cortical terminals were of the *en passant* and *terminaux* type, but were significantly smaller than the cortico-thalamic (average major diameter of 1.08 µm; range: 0.18 µm<diameter<4.2 µm), consistent with findings in other systems [21].

Thalamo-cortical terminals were polymorphic, of variable size (Fig. 9H) but fell into at least two distinguishable groups by bouton size. All layers contained approximately similar proportions of small (69%) and large (31%) boutons. Cluster analysis revealed at least two distinct groups that differed in size (p<0.00001), in both superficial and middle-deep layers (Fig. 9I). However, the average size of boutons was significantly smaller in the superficial (I-IIIa) than in the middle-deep (IIIb-VI) layers (p = 0.002; Fig. 9H), and both large and small boutons in the superficial layers were significantly smaller than the corresponding group of terminals in the middle-deep layers (p = 0.0009).

Having estimated that the majority of thalamo-cortical boutons terminating in the superficial layers were CB+, whereas most of the boutons in the middle-deep layers were PV+, one could assume that CB+ terminals were generally smaller than PV+ terminals. However, stereological analysis and comparison of the CB+ and PV+ bouton sizes in each layer revealed that this was not the case. Both CB+ and PV+ thalamo-cortical boutons in the superficial layers were smaller than the CB+ and PV+ boutons in the middle-deep layers (p = 0.0005; mean diameter of superficial boutons±SEM = 0.91±0.01; mean diameter of middle-deep boutons±SEM = 1.21±0.02).

Another significant difference between terminations in the superficial versus the middle-deep layers of prefrontal cortex was the area covered by single fibers. Thalamic axonal terminations were more widespread in the superficial layers, and in some instances the same axon could be traced horizontally for more than 1 mm in a coronal section, especially in layer I. Such axons often traversed the borders between cortical columns and even neighboring cortical areas (Fig. 9F). On the other hand, axons in the middle-deep layers were shorter, their terminations were more focused, and they were restricted to small columns, forming clusters (313<width<1,438 µm) within each cortical target (Fig. 9G).

#### Relationship of thalamo-cortical projections to neurochemical classes of inhibitory neurons in prefrontal cortex

**Figure 10:**
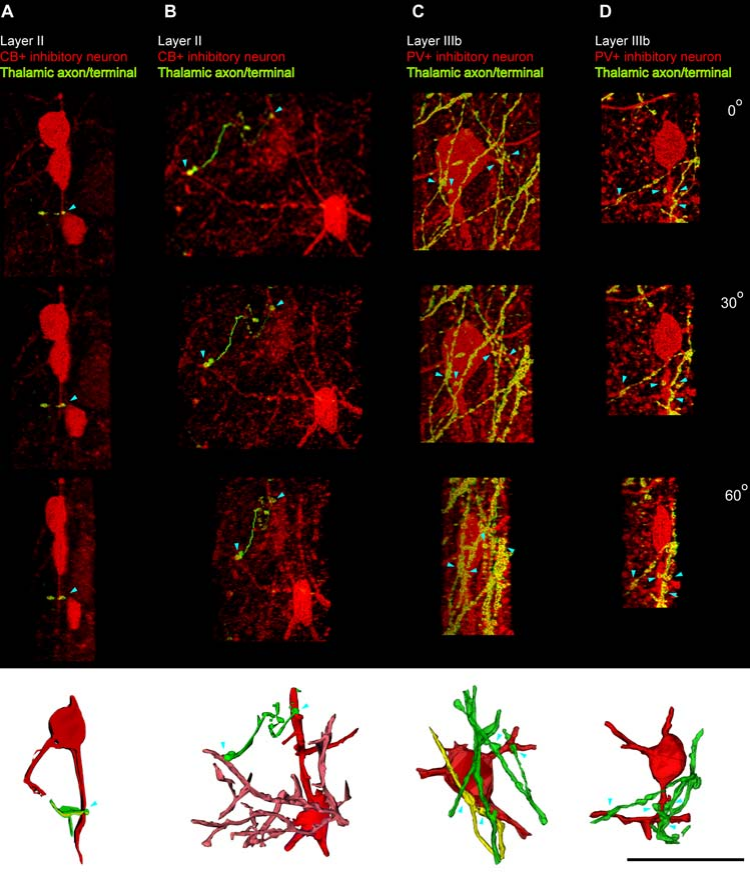
Appositions of laminar-specific thalamo-cortical terminals with local inhibitory neurons labeled with CB and PV in the prefrontal cortex. The first three images in each column are incrementally rotated by 30° around their Y axis, and the last row of renderings shows three-dimensional reconstructions of apposed neurons (red or pink) and fibers (green or yellow) in the images directly above. *Columns A, B,* Some thalamo-cortical terminals in layer II of area 32 (yellow) that are anterogradely labeled with green fluorescent tracer and also express CB (red) were closely apposed to CB+ local inhibitory neurons, likely forming synapses (blue arrowheads). *Columns C, D,* Some thalamo-cortical terminals in layer IIIb of area 9 (yellow) that are anterogradely labeled with green fluorescent tracer and also express PV (red), were closely apposed to local inhibitory neurons labeled for PV, and also form close appositions and possible synapses (blue arrowheads). Scale bar: 50 µm.

Thalamo-cortical projections terminating in distinct layers of prefrontal cortices encountered a microenvironment with distinct neurochemical classes of inhibitory neurons, as summarized in Figure 7F, G. As shown in previous studies [36], [37] and confirmed here, inhibitory neurons labeled with CB are found predominantly in the superficial layers of prefrontal cortex, whereas inhibitory neurons labeled with PV are found mostly in the middle-deep layers. Axons from the thalamus labeled with tracer and CB terminated within the superficial layers of prefrontal cortex, where they intermingled with local CB inhibitory neurons (Fig. 9A–C), and in some cases were closely apposed to them, likely forming synapses (Fig. 10A, B). On the other hand, axonal projections from the thalamus labeled with tracer and PV intermingled with PV inhibitory neurons in the middle-deep layers of prefrontal cortex (Fig. 9D, E), and were also closely apposed to them, forming possible synapses (Fig. 10C, D).

In summary, these findings indicate that pathways from the ventral anterior thalamic nucleus to prefrontal cortex are specialized. One pathway originated preferentially from CB+ thalamic neurons and targeted preferentially the superficial layers of prefrontal cortex, mostly through small terminals, consistent with a modulatory pathway. Another pathway originated preferentially from PV+ thalamic neurons and targeted preferentially the middle layers of prefrontal cortex, through generally larger terminals, consistent with a driving pathway. The two pathways originating from PV+ and CB+ thalamic neurons innervated cortical layers where PV and CB inhibitory neurons were most prevalent, respectively.

## Discussion

### Dual circuits link the prefrontal cortex with the ventral anterior nucleus

We present novel evidence on the architecture of two parallel reciprocal circuits between prefrontal cortex and the ventral anterior nucleus, which are distinguished by remarkable neurochemical and structural specificity. The high degree of segregation of these pathways is consistent with differences in neural dynamics elicited by their activation, and suggests complementary roles in the communication between the thalamus and prefrontal cortex.

**Figure 11:**
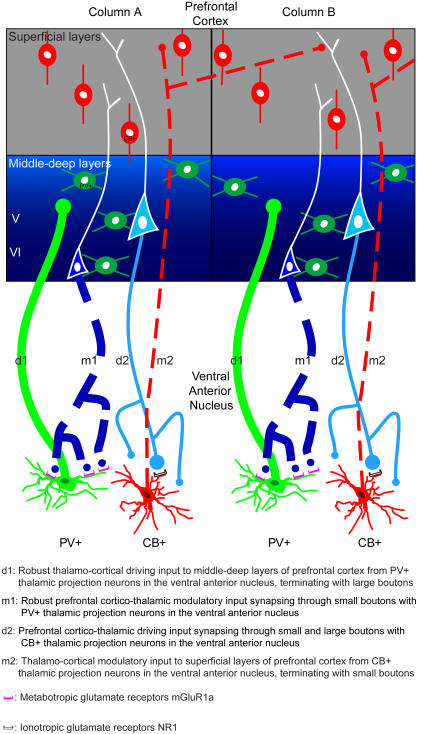
Schematic diagram summarizing the features of reciprocal driving and modulatory pathways linking the prefrontal cortex with the ventral anterior nucleus. The thickness of the lines, connecting thalamus and cortex, indicates the strength of the projection. The size of the dot indicates the size of the terminals. Solid lines (d1 and d2) represent driving projections, and dotted lines (m1 and m2) represent modulatory projections. Green represents PV+ labeling and red CB+ labeling. There were two parallel reciprocal circuits between the prefrontal cortex and ventral anterior nucleus. One originated mostly from PV+ thalamic projection neurons (green, bottom panels), and terminated focally as large boutons in the middle-deep layers (IIIb–VI, center blue panels) of the prefrontal cortex (d1). In turn, presumed layer VI neurons projected (m1) and terminated as numerous small boutons that formed synapses mainly with PV+ thalamic projection neurons (green, bottom panels), enriched with metabotropic receptors (mGluR1a, purple). The other circuit originated from CB+ thalamic neurons and sent widespread projections (m2) mainly to the superficial layers (I–IIIa) of the prefrontal cortex (grey), and terminated mostly as small terminals traversing the borders of neighboring regions and in association with the apical dendrites of neighboring layer V neurons. In turn, a prefrontal pathway (d2) established synapses through many small and fewer large boutons mainly on CB+ thalamic projection neurons (red) with ionotropic receptors (NR1, white). The PV+ thalamo-cortical pathway (d1) terminated mostly in the middle-deep layers, which were also rich with PV+ local inhibitory neurons (green spheres). In contrast, the CB+ thalamo-cortical pathway (m2) terminated in the superficial layers, which were rich in CB+ local inhibitory neurons (red spheres).

As summarized in Figure 11, one circuit originated mostly from PV+ thalamic neurons, projected focally mostly through large boutons to the middle layers of prefrontal cortex (Fig. 11, d1), and was innervated by small prefrontal terminals (Fig. 11, m1). The other circuit originated from CB+ thalamic neurons, and terminated broadly mostly through small terminals in the superficial layers of prefrontal cortex (Fig. 11, m2), with only sparse terminations in other layers. The CB+ thalamic neurons were innervated by prefrontal axons that terminated as a mixed population of mostly small, but also a significant proportion of large boutons (Fig. 11, d2).

### Specificity of cortico-thalamic pathways

Further specificity in the parallel cortico-thalamic pathways was revealed by synapses between large prefrontal boutons and CB+ thalamic projection neurons with perisynaptic ionotropic glutamate receptors, NR1 (Fig. 11, d2). In contrast, small prefrontal boutons formed synapses mainly with PV+ thalamic projection neurons with metabotropic glutamate receptors, mGluR1a (Fig. 11, m1). We found no evidence of large boutons forming synapses with mGluR1a, even though some large terminals established synapses with PV+ dendrites, in agreement with previous studies [38]. These glutamate receptor subtypes were expressed by both CB+ and PV+ thalamic neurons but their perisynaptic localization correlated with the size of cortico-thalamic terminals.

Cortical glutamatergic projections provide the predominance of excitatory synaptic inputs in the thalamus [1], [2], shown specifically for the ventral anterior nucleus [39]. This evidence suggests that cortico-thalamic axons have a key role in controlling the firing patterns of thalamo-cortical relay neurons. Large terminals evoke all-or-none excitatory postsynaptic potentials (EPSPs) that show paired-pulse depression and are antagonized by NMDA receptor blockers, but have no metabotropic glutamate receptor components [14]. The present findings provide the structural basis for the physiological findings, and further indicate the specificity of this pathway in targeting CB+ thalamic dendrites enriched with fast ionotropic receptors. Driving input, presumably originating in cortical layer V, resembles projections from peripheral sensory systems, or from subcortical movement-related centers to the thalamus. All of these projections terminate in clusters of large boutons, form synapses with relay cells, and interact with ionotropic glutamate receptors in the thalamus [8], [9], [12], [15], [40]. The cortico-thalamic pathway originating from large layer V neurons also innervates motor centers in the brainstem and spinal cord, and is presumed to carry a copy of motor commands to the thalamus [41]–[43]. In contrast, small axonal boutons, evoke small, graded EPSPs showing paired-pulse facilitation with NMDA, AMPA and mGluR1 receptor components [14], [16], [17], [44]–[46]. Our findings indicate further specificity in this pathway through preferential synapses with PV+ thalamic dendrites with perisynaptic slow metabotropic receptors (mGluR1). This modulatory input, which may originate from cortical layer VI, is more abundant and widespread, and is thought to modify the driving signal [13]–[15], [22], [47], [48]. These findings support the hypothesis that a driver input, acting through large boutons, conveys essential, raw information with maximal impact reflected as large EPSPs. In contrast, modulatory cortico-thalamic input through small boutons may underlie sustained EPSPs [15].

Interestingly, unlike other cortices, prefrontal cortico-thalamic axons also innervate the inhibitory thalamic reticular nucleus in a similar manner, through small and large boutons resembling modulatory and driving inputs, respectively [27]. In addition, prefrontal cortico-thalamic axons target local inhibitory neurons in the ventral anterior nucleus [39], potentially affecting the balance of excitation/inhibition.

#### Significance of bouton size

Large cortico-thalamic boutons had more mitochondria and more synaptic vesicles than small boutons, in proportion to their size [28], [49]–[51]. Moreover, the vesicles in large boutons were arranged in distinct clusters, resembling readily releasable and reserve pools. Large boutons are more likely to undergo multivesicular release and could be more efficient in activating their targets [52], [53]. Large boutons may be present in highly active networks, consistent with their increased mitochondrial content, which is activity dependent [54], and their large axon caliber likely allows fast transmission.

Several studies have linked bursting frequency in thalamic nuclei with the ratio of small to large cortical terminals. The ratio is higher in high-order than in first-order thalamic nuclei, potentially leading to lower spontaneous activity and more bursting in high-order nuclei [55]–[59]. In our material, the ratio of small to large boutons in the ventral anterior nucleus was 13:1, smaller than in the high-order pulvinar nucleus (24:1) but larger than the first-order dorsolateral geniculate nucleus (5:1) [55], [60].

#### Laminar origin of cortico-thalamic pathways and size of boutons

We confirmed the dual and nearly equal origin of prefrontal projections from layers VI and V to the ventral anterior nucleus [26]. Several studies have associated the prevalent small terminals in the thalamus with origin in cortical layer VI, and the sparser large terminals with cortical layer V. These conclusions could be reached with confidence in systems that issue projections exclusively from layer VI, as in the projection from primary visual cortex to the dorsolateral geniculate nucleus [61], or exclusively from layer V, as in the projections from visual cortices to the lateral posterior nucleus and the inferior pulvinar [61], [62]. However, in the vast majority of cortico-thalamic systems with dual origin in layers VI and V, the laminar origin of axons giving rise to small or large boutons can be inferred only by analogy with the above systems. Such inference, however, is complicated here by the presence of both small and large boutons on single branches of cortico-thalamic axons, seen also in other thalamic nuclei [13], [27]. These findings indicate that significant numbers of cortico-thalamic neurons produce both endings so that it's not possible to associate projections from all neurons in layer V with driver-like transmission.

### Specificity of thalamo-cortical pathways

In an analogous pattern, the thalamo-cortical circuits also appeared to be organized into two systems, but had the reverse organization (Fig. 11). A driver-like pathway terminated in the middle-deep layers of prefrontal cortex, consisting mainly of large PV+ terminals (Fig. 11, d1), and a modulatory pathway terminated in the superficial layers, containing mainly smaller CB+ terminals (Fig. 11, m2). Thalamo-cortical projections targeting mostly the middle or superficial cortical layers have been reported for diverse thalamic nuclei [2], [11], [19], [21], [25], [62]–[64]. Evidence based on sensory systems, led to the hypothesis that there are two distinct assemblies of thalamic projection neurons. A core thalamic system of PV+ neurons acts as a relay, and a CB+ matrix appears to be engaged in intrathalamic corticocortical communication through widespread projections to superficial cortical layers [19]. These pathways correspond, respectively, to driver and modulatory projection systems [8], [11]. Our findings suggest that this pattern applies to the prefrontal cortex as well, and may be general across cortical systems. The distinct thalamic populations can be topographically segregated and structurally different, like the giant CB+ neurons in the pulvinar [19], [21], or intermingled and similar, as seen here for the ventral anterior and in other thalamic nuclei [18]. Further studies are necessary to determine if there are comparable interactions of these pathways with, respectively, ionotropic and metabotropic receptors as has been suggested [15], [65].

Alternatively, experimental and modeling work suggests that both drivers and modulators in the cortex interact solely with ionotropic receptors [23]. In this model, excitatory driving input reduces local inhibition on the targets, strengthening the excitatory effect. On the other hand, excitatory modulatory input increases both excitation and inhibition locally, differentially adjusting the gain of neurons. Such a scenario is possible, in view of the relative laminar segregation of large and small terminals of thalamo-cortical pathways, and their interaction with functionally distinct and laminar-specific neurochemical classes of inhibitory neurons [36], [37]. Thus, the excitatory PV+ thalamic projections terminated mostly in the middle cortical layers, among neurons that also express PV, but are inhibitory neurons found predominantly in the middle-deep layers of the cortex. Cortical PV+ neurons innervate perisomatic sites of neighboring pyramidal neurons, exercising strong inhibitory control (reviewed in [66]). On the other hand, excitatory CB+ thalamic projections terminated most densely in the superficial cortical layers, among CB+ inhibitory neurons, which are most prevalent in the superficial cortical layers, and are known to innervate the distal dendrites of neighboring neurons [66], exercising a modulatory influence. While the majority of thalamo-cortical terminals form synapses with excitatory pyramidal neurons, a significant number establish synapses with local inhibitory neurons [67]. In addition, in rats approximately one third of spines of cortical pyramidal neurons that form synapses with thalamic terminals also form synapses with local inhibitory neurons [68]. The potential interactions of thalamic pathways with functionally distinct neurochemical classes of cortical inhibitory neurons in the primate model used here may provide a mechanism for shifts in neural dynamics [69]–[71].

### Functional implications for transthalamic cortico-cortical communication

The ventral anterior thalamic nucleus has a special relationship with the prefrontal cortex and with the basal ganglia, associated in common with emotional, cognitive, and motor functions [25], [26]. The delicate balance of driving and modulatory inputs and outputs is disrupted in diseases, such as schizophrenia, where fewer PV+ thalamo-cortical varicosities are reported in the middle layers of prefrontal cortex [72]. Disruption of a strong linkage of lateral and medial prefrontal areas with the ventral anterior nuclei and the basal ganglia could lead to cognitive and emotional deficits, including inability to initiate speech, or readily change facial expression in emotional situations, associated with Parkinson's disease [73]–[76]. It has been shown that pallidal hyperactivity over inhibits the motor thalamus, including the ventral anterior nucleus, producing thalamo-cortical dysrhythmia that generates the clinical Parkinsonian symptoms [77]. The dense dopaminergic innervation of the ventral anterior nucleus and the prefrontal cortex further support the potential role of these structures in these diseases [78]–[80]. In addition, a recent functional imaging study indicated that the ventral anterior thalamus is excessively connected with the prefrontal cortex in autistic people [81], highlighting yet another abnormality in this circuit [82], [83].

The novel quantitative evidence presented suggests that the two parallel circuits that link the prefrontal cortex with the ventral anterior nuclei have complementary functions. A pathway from one circuit may relay focal information to the middle cortical layers, and a pathway from a parallel circuit may mediate complex modulatory interactions through widespread superficial layer projections. The latter innervate the apical dendrites of neighboring layer V pyramidal neurons, suggesting that neurons outside an active cortical zone may increase their gain and ultimately their output to the thalamus and back to the cortex, transmitting excitation from one site to another [9], [19], [25], [84]. In concert with cortico-cortical connections, the two distinct circuits through prefrontal cortex and the ventral anterior thalamic nucleus could link structures associated with perception, emotion and action.

Further, the dual thalamo-cortical pathways likely interact with distinct classes of inhibitory neurons in the cortex, and the dual cortico-thalamic pathways interact with the inhibitory thalamic reticular nucleus [27] and local inhibitory neurons in the thalamus [2], [39] providing a remarkable reciprocity in the connections with excitatory and inhibitory systems. The prefrontal cortical projections to the reticular nucleus in rhesus monkeys similarly terminate as small, modulatory-like, and large, driver-like boutons, and are uniquely widespread, extending into the sensory sectors of the nucleus, and may affect the flow of sensory information from the thalamus to the cortex [27], [85]. These connections have a critical role in controlling the firing patterns of thalamocortical relay neurons, and may allow selection of relevant information and override distractors.

The interface of pathways linking the prefrontal cortex with excitatory and inhibitory systems could alter the balance of excitation and inhibition affecting rhythmic oscillations. Differential engagement of the parallel cortico-thalamic circuits may mediate shifts to different functional states, ranging from active attention in the alert state needed for cognitive operations, to a drift to drowsiness and sleep. Disturbances in these functional states of everyday life are seen in several psychiatric diseases [72], [77], [86], and may reflect a change in the intricate balance of neurochemically-specific pathways that link the thalamus with prefrontal cortex.

## Materials and Methods

### Model system

Rhesus monkeys (*Macaca mulatta*) were used as a non-human primate model system to study the interactions of the high-order association prefrontal cortices with the thalamus for several reasons. The prefrontal cortex is highly developed in primates, occupying a large proportion of the cortex. The thalamus of macaque monkeys has a dual and complementary projection system through relay cells that are positive for the calcium binding proteins calbindin or parvalbumin, which differs from non-primate species [18], [19], [87]. Finally, in the rhesus monkey prefrontal projections to the thalamus have a dual origin in layers VI and V, with substantial projections from layer V in comparison with other cortices [88]. About half of prefrontal projection neurons directed to the ventral anterior thalamic nucleus, in particular, originate in layer V, rendering this system an ideal model for this study [26].

### Animal and tissue preparation

Experiments were conducted on 10 young adult rhesus monkeys (2–3 years of age) in accordance with National Institutes of Health Guide for the Care and Use of Laboratory Animals (publication 80–22 revised, 1996). The experiments were approved by the Institutional Animal Care and Use Committee at Boston University School of Medicine, Harvard Medical School, and New England Primate Research Center. Procedures were designed to minimize animal suffering and reduce their number.

We calculated the stereotaxic coordinates for each injection in three dimensions using the interaural line as reference after obtaining three-dimensional scans of each brain using magnetic resonance imaging (MRI) in anesthetized animals, as described previously [27]. One week after the MRI, the monkeys were anesthetized with ketamine hydrochloride (10–15 mg/kg, i.m.) followed by gas anesthetic (isoflurane), until a surgical level of anesthesia was accomplished. Surgery was performed under aseptic conditions while heart rate, muscle tone, respiration, and pupillary dilatation were closely monitored. The monkeys were placed in a stereotaxic apparatus, and a small region of the cortex was exposed.

**Figure 12:**
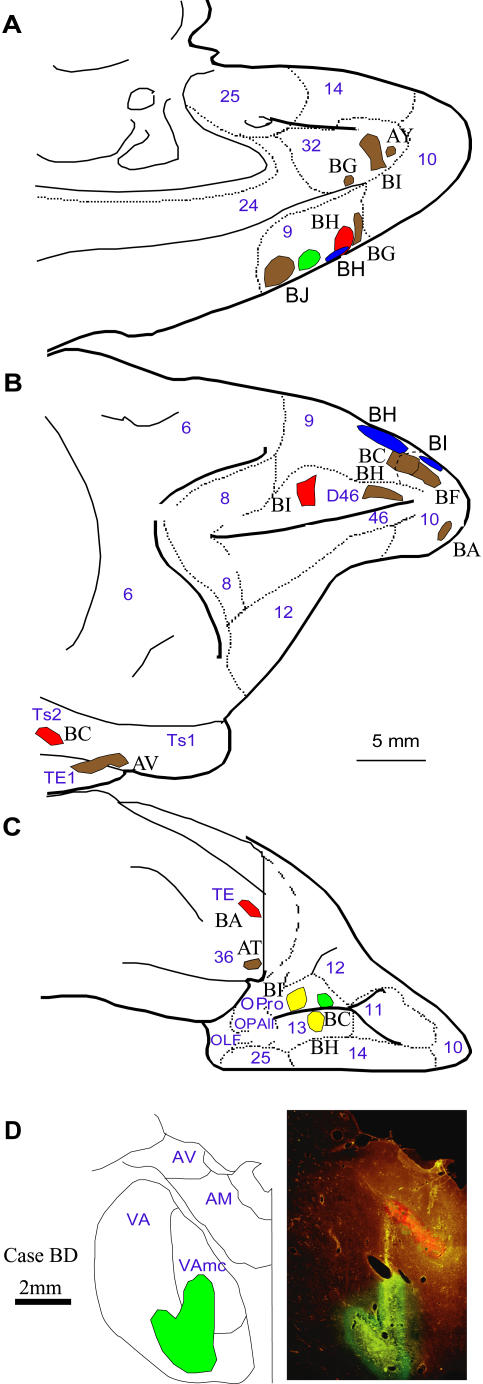
Composite of cortical and thalamic injection sites. Cortical injection sites shown on the *A,* medial, *B,* lateral, and *C,* basal surfaces of the brain of the rhesus monkey. *D,* Representative coronal section through the thalamus (photomicrograph on the right column and corresponding outlines on the left column). The red fluorescence in the anterior medial thalamic nucleus is from a fluororuby injection, which was not used in this study. Case names appear in black; Names of areas and nuclei in A-D are in blue. Abbreviations: AM: anterior medial nucleus; AV: anterior ventral nucleus; VA: ventral anterior nucleus; VAmc: ventral anterior nucleus, pars magnocellularis; OLF: olfactory cortex; OPAll: orbital periallocortex; OPro: orbital proisocortex.

We injected anterograde, retrograde or bidirectional tracers in prefrontal areas 9, 10, 46, 13, and 32, and the ventral anterior thalamic nucleus (for map of injection sites, see Fig. 12A–D; Table S1). Using a microsyringe (Hamilton, 5 µl) mounted on a microdrive, we injected the bidirectional tracers biotinylated dextran amine (BDA; 10% solution, 6–10 µl, 10 kDa; Invitrogen, Carlsbad, CA), fluoro-emerald (FE; dextran fluorescein, 10% solution, 3–5 µl, 10 kDa; and in some cases a mixture of 3 and 10 kDa; Invitrogen), fluoro-ruby (FR; dextran tetramethylrhodamine, 10% solution, 3–4 µl, 10 kDa; and in some cases a mixture of 3 and 10 kDa; Invitrogen), and Lucifer yellow (LY; dextran, Lucifer yellow, anionic, lysine fixable; 10% solution, 3–4 µl, 10 kDa; Invitrogen). In some cases, we injected the retrograde tracer fast blue in the ventral anterior nucleus (FB; 1% solution, volume of 0.3 µl, 379.84 Da; Sigma, St. Louis, MO), or placed it on the surface of the cortex to retrogradely label thalamic projection neurons to the superficial layers. In agreement with previous reports utilizing surface application of tracers, examination of the spread of the tracer in coronal sections showed limited spread restricted to the superficial layers, especially layer I [18], [89]. In each case, the tracers were injected in two penetrations (half the quantity in each penetration) and, for the cortical injections, at a depth of 1.2–1.6 mm below the pial surface. After injection, the needle was left in place for 10–15 min to allow the dye to penetrate at the injection site and prevent upward suction of the dye during retraction of the needle.

The tracers BDA, FE, FR and LY are excellent anterograde tracers that label the entire extent of axons and boutons. In the 3 kDa molecular weight they also act as retrograde tracers, and were used to label projection neurons. The extent of the labeling is restricted to the cell bodies and proximal dendrites of projection neurons and the tracer does not enter axon collaterals [90]–[92]. The large boutons seen in the ventral anterior nucleus, therefore, could not have belonged to collateral axons from retrogradely labeled neurons or from other thalamic nuclei that project to the prefrontal cortex, since dorsal thalamic nuclei are not interconnected. To study pathways in the anterograde direction, we used mainly the 10 kDa form of BDA, FE, or FR, which is optimal for anterograde but not retrograde labeling [91], [92]. Comparison of the labeled bouton population in these cases and in cases where a cocktail of 3 and 10 kDa FE, or FR were used revealed no differences. In addition, small and large labeled boutons from prefrontal projections were similar to anterograde labeling in the thalamic reticular nucleus [27], which does not project to the cortex. Trans-synaptic transport of any of these tracers has not been observed.

After a survival period of 18 days, the animals were anesthetized, transcardially perfused with 4% paraformaldehyde and 0.2% glutaraldehyde, and the brains were removed from the skull, cryoprotected in graded solutions of sucrose (10–30%), frozen, and cut on a freezing microtome in the coronal plane at 40 or 50 µm, as described previously [27]. In cases with injection of fluorescent tracers, one series was mounted on glass slides, coverslipped, and used to map labeled neurons and terminals.

### Examination of cortico-thalamic pathways

#### Experimental design

Figure 1A summarizes our experimental approaches. We first studied the global system of cortico-thalamic pathways at their origin in prefrontal cortex and their termination in the ventral anterior nucleus, as shown in Figure 1A. We investigated the topography of projection neurons in prefrontal cortices that were labeled retrogradely from the ventral anterior nucleus, and determined their density and laminar origin in layers V and VI. We then determined the distribution of terminals in the ventral anterior nucleus that were labeled in the anterograde direction from prefrontal cortices. Using material from the latter cases we then conducted a more detailed analysis of the ultrastructural features of cortical terminals in the ventral anterior nucleus and identified the neurochemical characteristics of their postsynaptic targets, as described below in detail.

#### Histochemical and immunohistochemical procedures-brightfield and fluorescence microscopy

In experiments with tracer injections, one series of sections was processed to visualize boutons and labeled neurons, using standard bright-field or fluorescence immunohistochemical protocols, as described previously [27]. Briefly, in cases with BDA injections, free-floating sections were rinsed in 0.01 M PBS, pH 7.4, and incubated for 1 hour in an avidin-biotin HRP complex (AB-Kit, Vector laboratories, Burlingame, CA; diluted 1:100 in 0.01 M PBS with 0.1% Triton X). The sections were then washed and processed for immunoperoxidase reaction using diaminobenzidine (DAB, Zymed laboratories, San Francisco, CA). Sections were then mounted, dried and coverslipped with Permount or Entellan (Merck, Whitehouse, NJ).

In some cases with FE, FR and LY injections in the prefrontal cortex, we used polyclonal antibodies (anti-fluorescein/Oregon Green, anti- tetramethylrhodamine and anti-LY, 1:800; Molecular Probes) to convert the fluorescent tracers and visualize label by the peroxidase-catalyzed polymerization of DAB. To avoid cross-reaction with the BDA we used non-biotinylated polyclonal anti-rabbit secondary antibody followed by tertiary anti-rabbit solution (PAP method, 1:200; Sternberger Monoclonals, Lutherville, MD).

#### Electron microscopy: Double and triple labeling pre-embedding immunohistochemistry

To view presynaptic label of axon terminals from prefrontal areas in the ventral anterior nucleus, and determine whether they synapse with PV or CB labeled dendrites, and the type of glutamate receptors on postsynaptic sites, we used multiple labeling procedures. We selected two types of glutamate receptors based on their abundance and distribution in the mammalian thalamus. Previous studies have shown that the ionotropic NR1 and metabotropic mGluR1 glutamate receptor subtypes are the most abundant in the dorsal thalamus, and the ventral anterior nucleus specifically [93], [94]. They also seem to be the receptors most likely activated by cortico-thalamic afferents [14], [16], [94], [95].

Sections treated to view axonal terminals from prefrontal cortex (labeled with BDA, FE, FR, or LY) for brightfield microscopy were then processed for electron microscopy. We used the protocols described above, but Triton X-100 concentration in all solutions was reduced to 0.025%. To determine the relationship of labeled boutons from prefrontal areas to ventral anterior thalamic neurons, we double labeled sections using antibodies against CB or PV (1:2000, mouse monoclonal; Chemicon, Temecula, CA) and pre-embedding immunohistochemistry and visualized label with gold-conjugated anti-mouse secondary antibodies (1:50; gold particle diameter, 1 nm; Amersham Biosciences, Piscataway, NJ). To prevent diffusion of the gold particles, tissue was postfixed in a microwave with 6% glutaraldehyde, after quick rinses in PBS. Labeling was intensified with the use of the Amersham Biosciences silver enhancement kit (IntenSE).

We used triple labeling pre-embedding immunohistochemistry to investigate the type of glutamate receptors present postsynaptically on CB or PV thalamic neurons that form synapses with prefrontal terminals labeled with BDA. Prefrontal terminals labeled with BDA were visualized with DAB, as described previously, and then sections were incubated in Avidin-Biotin blocking kit solutions (Vector Labs) to block free binding sites. Glutamate receptors were labeled with antibodies raised in rabbits against ionotropic NMDA receptor 1 (NR1; 1:200, Chemicon) or metabotropic receptor mGluR1a (1:800, Chemicon) and visualized with gold-conjugated anti-rabbit secondary antibodies (1:50; gold particle diameter, 1 nm; Amersham Biosciences), as described above. Sections treated for NR1 or mGluR1a receptors were also co-incubated overnight with CB or PV (as above) and processed for immunoperoxidase reaction using tetramethylbenzidine (TMB; 5-15 min incubation; 0.005% TMB in 100% ethanol, 5% ammonium paratungstate, 0.004% NH_4_Cl, 0.005% H_2_O_2_ in 0.1 M PB, pH: 6), which produces a reaction product of grainy rods that is distinct from the uniform reaction product of DAB.

Tissue sections with label were mounted on slides and quickly viewed under the light microscope, and images were captured with a CCD camera. Small blocks of sections with anterograde, NR1 or mGluR1a and CB or PV label were cut under a dissecting microscope, postfixed in 1% osmium tetroxide with 1.5% potassium ferrocyanide in PB, washed in buffer (PB) and water, and dehydrated in an ascending series of alcohols. While in 70% alcohol, they were stained with 1% uranyl acetate for 30 min. Subsequently they were cleared in propylene oxide and embedded in araldite at 60°C. Serial ultrathin sections (50 nm) were cut with a diamond knife (Diatome, Fort Washington, PA) using an ultramicrotome (Ultracut; Leica, Wein, Austria) and collected on single slot grids to view with a transmission electron microscope (100CX; Jeol, Peabody, MA).

#### Intracellular injections

To determine whether prefrontal axons terminated on PV or CB labeled dendrites that differed in size, we injected thalamic projection neurons intracellularly to fill and measure the dendritic tree. These thalamic neurons were then reconstructed in three dimensions to measure the diameters of their proximal and distal dendrites. For this analysis we considered as proximal the primary and secondary dendrites. We considered all other parts of the dendritic tree distal. The brains were removed from the skull and stored in a fixative solution containing 4% paraformaldehyde and 0.05% sodium azide in 0.1 M PB, pH 7.4. Blocks of tissue containing the ventral anterior nucleus were dissected and cut on a vibratome in the coronal plane at 300 µm. Sections that included retrogradely labeled fluorescent thalamic projection neurons connected with the prefrontal cortex were processed for parvalbumin or calbindin immunofluorescence (see previous paragraph) and positioned under a nylon mesh in a submersion type slice recording chamber (Harvard Apparatus, Holliston, MA) filled with PBS. The chamber was placed on the stage of an infrared-differential interference contrast (IR-DIC) microscope (Olympus BX 51 WI), connected to a personal computer via an infrared CCD camera (QICAM-IR, Q-Imaging). Double labeled cells were identified under fluorescence illumination and filled under infrared or fluorescent light with Lucifer Yellow CH, lithium salt (LY, Invitrogen: 1% in 0.05 M Tris buffer, pH 7.5) or Alexa Fluor 488 hydrazide sodium salt (Invitrogen: 1% in 0.05 M Tris buffer, pH 7.5) by continuous negative current (9–50 pA) until the dendritic tree fluoresced brightly.

### Analysis of thalamo-cortical pathways

#### Experimental design

The experimental approach is summarized in Figure 1B. We mapped the distribution and estimated the density of CB+ and PV+ thalamic projection neurons in the ventral anterior nucleus that project to prefrontal cortex. In addition, we quantitatively mapped the laminar distribution and measured the size of CB+ and PV+ terminals in the prefrontal cortex originating from the ventral anterior nucleus. We also examined the laminar relationship of thalamic axonal terminations in the prefrontal cortex to local inhibitory neurons labeled with CB+ or PV+, as described in detail below and shown in Figure 1B.

#### Histochemical and immunohistochemical procedures - brightfield and fluorescence microscopy

CB and PV are expressed in distinct classes of projection neurons in the thalamus and distinct neurochemical classes of inhibitory neurons in the cortex. To identify each of these classes of projection neurons in the ventral anterior nucleus and local inhibitory neurons in the prefrontal cortex, we used antibodies against the calcium binding proteins CB and PV. The tissue was rinsed in 0.01 M PBS, pH 7.4, followed by 10% normal goat serum, 5% bovine serum albumin, and 0.1% Triton X-100 in 0.01 M PBS blocking solution for 1 h and incubated for 1 day in primary antibody for CB or PV (1:2000, mouse monoclonal). The sections were rinsed in PBS, incubated for 1 h with goat anti-mouse IgG conjugated with the fluorescent probes Alexa Fluor 488 (green) or 568 (red; 1:100; Invitrogen) and thoroughly rinsed with PBS. In some cases, a biotinylated secondary antibody and an avidin–biotin–peroxidase kit was used to label CB+ or PV+ structures with DAB. Since in most cases there was also a BDA injection in one of the brain hemispheres, we used an Avidin-Biotin blocking kit (Vector Labs, Burlingame, CA) at the beginning of the process, to saturate biotin binding sites from BDA. In other cases we labeled CB+ or PV+ structures with DAB using the PAP method and non-biotinylated secondary antibodies. To exclude nonspecific immunoreactivity, we performed control experiments, with sections adjacent to those used in the experiments described above. These included omission of the primary antibodies and incubation with all secondary or tertiary antisera. All control experiments resulted in no immunohistochemical labeling.

### Stereological analysis

We mapped the architecture of thalamic nuclei and the prefrontal cortex from Nissl and acetylcholinesterase-stained sections matched to sections that were immunohistochemically labeled. The nomenclature of the thalamus is according to the map of Jones [2], as modified from the map of Olszewski [96], and for the prefrontal cortex is according to the map of Barbas and Pandya [97], modified from the map of Walker [98]. In limbic areas 24, 32 and 13, the layers were not clearly delineated. We considered the acellular gap between the deep (V and VI) and superficial (I and II/IIIa) layers as the middle layers for those cortices.

#### Bouton population analysis

We analyzed anterograde labeling in the ventral anterior nucleus and in different layers of the prefrontal cortex at high magnification (1000x) using unbiased stereological methods [99], as described previously [27]. Briefly, the systematic, random sampling fraction was 1/50 of the total volume of the region studied and resulted in measuring the morphological characteristics of >4000 labeled bouton profiles in each case. The morphological characteristics measured were the minor and major diameter, perimeter, and surface area.

#### Neuron and bouton density estimate

We estimated the numbers of prefrontal projection neurons in layers V and VI, the numbers of CB+ and PV+ thalamic projection neurons, the quantitative laminar distribution of CB+ and PV+ prefrontal inhibitory neurons, as well as anterogradely labeled boutons coming from the ventral anterior nucleus and terminating in the prefrontal cortex and vice-versa, using the unbiased stereological method of the optical fractionator [99], [100] with the use of specific software (StereoInvestigator; Microbrightfield, Williston, VT, USA), as described previously [27]. The advantage of this method is that it is not affected by tissue shrinkage [99]–[101]. In using the StereoInvestigator software, the height of each section is measured prior to counting the particles of interest. To ensure unbiased estimate of objects counted, the method uses a guard zone at the bottom and top of each section to correct for objects plucked during sectioning, so the disector thickness is always smaller than the thickness of the section.

The sampling fraction was 1/16 of the total volume of each area examined for bouton number estimation, and 1/50 for neuronal numbers, and was determined in pilot studies using exhaustive sampling and progressive means analysis so that final estimates had a standard error ≤10%. The use of uniform random sampling ensured that every part of each area examined had the same chance of being included in the sample. Large and small boutons were measured separately and as a single population. In the second case they were systematically distinguished based on the results of the bouton population analyses (see previous section). The estimated numbers of neurons or boutons and the volumes of the corresponding areas or nuclei estimated with the Cavalieri method were divided to assess the density of label in each case. We normalized data by expressing the density of neurons or large and small boutons as a percentage of the total density of all labeled neurons or boutons in the prefrontal cortex or the ventral anterior nucleus in each case.

### Three-Dimensional reconstruction and imaging

#### Light and confocal laser microscopy

To compare the topography and distribution of anterograde and retrograde labeling across cases and exhibit their overlap in the ventral anterior nucleus, we reconstructed in three dimensions the entire nucleus using the free, open source software Reconstruct [102]. With the aid of Neurolucida software (Microbrightfield, Williston, VT, USA), we traced the borders of the ventral anterior nucleus in all coronal sections used for stereological analysis, spanning the entire rostrocaudal extent of the nucleus. We then imported the outlines containing three-dimensional information about the topography of labeling in Reconstruct, aligned them, and generated three-dimensional models. As a result, all markers and traces were stereotaxically registered and superimposed on the three-dimensional models. To exhibit the distribution of large and small terminals in the ventral anterior nucleus originating in different prefrontal areas, we imported the outlines of sections and labeling in Reconstruct, color coded the bouton markers (large boutons, blue; small boutons, red) and generated three-dimensional maps.

For the analysis of thalamo-cortical projections from the ventral anterior nucleus to the prefrontal cortex, the distribution of projection neurons in layers V and VI and the distribution of CB+/PV+ local inhibitory neurons we viewed adjacent or double labeled sections under bright-field or fluorescence illumination, outlined the borders of areas and their layers, and traced labeled axons and terminations using the Neurolucida software. Deconvolution of images, with the aid of Autodeblur (Media Cybernetics, Silver Spring, MD), was necessary to eliminate the inevitable fluorescent signal halo and accurately assess whether fibers and terminals were double- or single-labeled. Previous attempts to use various brightfield double labeling methods did not allow quantitative analysis of all double-labeled thalamo-cortical fibers, because strongly labeled fibers and terminals appear black [103].

Images presented or used for analyses were captured at high resolution with a CCD camera mounted on an Olympus Optical BX51 microscope (Thornwood, NY) connected to a personal computer, using commercial imaging systems [MetaMorph version 4.1 (Universal Imaging Corporation) or Neurolucida]. Image stacks of several focal planes were acquired in each area of interest, resulting in pictures with high depth of field of 40 to 50 µm thick sections focused throughout their z-axis extent. Stacked images from adjacent serial sections were superimposed to highlight the differential distribution or overlap of retrogradely labeled neurons and anterogradely labeled boutons. Stereological measurements of the numbers of labeled fibers that contained exclusively small or large boutons or a mixed population were conducted on such images, as described previously [27]. Our samples included widely spaced sections (one every twenty) and fields of view, selected by a systematic random sampling method to minimize the likelihood of selecting varicose axons from the same parent branch.

Following intracellular injections, sections were viewed under high magnification at a confocal laser microscope (Olympus Fluoview) and stacks of images were captured. We applied three-dimensional deconvolution algorithms to images prior to analysis with the aid of Autodeblur. Only neurons that were well filled were included for analysis. The size (width) of proximal and distal dendrites was determined with the aid of ImageJ (NIH Research Services, Bethesda, MD). Photomicrographs were prepared with Adobe Photoshop (Adobe Systems, San Jose, CA), and overall brightness and contrast were adjusted without retouching.

#### Electron microscopy

We viewed at least 200 labeled boutons emanating from prefrontal axons from areas 46, 32, and 9 as well as neighboring unlabeled boutons forming synapses in the ventral anterior nucleus neurons at high magnification (10,000x) and photographed them throughout their entire extent (between 10 and 80 serial ultrathin sections) using exhaustive sampling. Film negatives were scanned, imported as a series in Reconstruct and aligned, as described previously [27]. Boutons, postsynaptic densities, and other postsynaptic structures (e.g., dendrites) were traced, reconstructed in three dimensions, and their volumes, surface areas, and diameters calculated. We used classic criteria for identifying synapses and profiles [28], [104], [105].

We used the single section disector method to estimate the size and number of vesicles contained in a random sample of labeled prefrontal cortico-thalamic boutons (n = 19). This method assumes that if an object that appears in one section has a major diameter much smaller than the section thickness it would rarely appear in the following sections [99]. The vesicles in our tissue had an average major inner diameter ∼27 nm, half the section thickness (50–60 nm). Moreover, vesicles were counted in 1 of every 3 sections to ensure that they were not overestimated by counting split vesicles. We also measured the inner diameter of 1/3 of the total population of vesicles and calculated their volume, assuming a spherical shape.

### Statistical analysis

Data were evaluated with Statistica (StatSoft, Tulsa, OK), through scatter and frequency distribution plots and K-means cluster analysis with parameters set to maximize initial between-cluster distances. We used x^2^ and Kolmogorov–Smirnov tests to examine bouton size distributions. We used ANOVA to test for differences among bouton populations and densities, and p values <0.01 were taken as statistically significant. Post hoc analyses using Bonferroni's/Dunn's (all means) was performed to identify possible differences between groups.

## Supporting Information

Figure S1 Prefrontal axonal terminations in the ventral anterior nuclei. Two-dimensional quantitative projection patterns in six representative coronal sections covering the whole rostrocaudal extent of the ventral anterior nucleus (dotted black outline) and its magnocellular part (black outline), shown in 3D-reconstruction (top, left). Boutons from prefrontal axons are represented by colored dots as follows: red, area 32; blue, area 9; green, area 10; orange, area 46; grey, orbital area 13. Each dot represents approximately 50 boutons.
(0.62 MB TIF)

Table S1 (0.03 MB DOC)

Text S1 (0.03 MB DOC)

Text S2 (0.04 MB DOC)

## References

[1] Steriade M, Jones EG, McCormick DA (1997). Thalamus-Organisation and function..

[2] Jones EG (1985). The Thalamus..

[3] Steriade M, Timofeev I (2003). Neuronal plasticity in thalamocortical networks during sleep and waking oscillations.. Neuron.

[4] Llinas RR, Steriade M (2006). Bursting of thalamic neurons and states of vigilance.. J Neurophysiol.

[5] Steriade M (2004). Local gating of information processing through the thalamus.. Neuron.

[6] Contreras D, Destexhe A, Terrence JS, Steriade M (1996). Control of spatiotemporal coherence of a thalamic oscillation by corticothalamic feedback.. Science.

[7] Steriade M, McCormick DA, Sejnowski TJ (1993). Thalamocortical oscillations in the sleeping and aroused brain.. Science.

[8] Guillery RW, Sherman SM (2002). Thalamic relay functions and their role in corticocortical communication: generalizations from the visual system.. Neuron.

[9] Sherman SM, Guillery RW (2002). The role of the thalamus in the flow of information to the cortex.. Philosophical Transactions of the Royal Society of London Series B-Biological Sciences.

[10] Jones EG (2002). Thalamic circuitry and thalamocortical synchrony.. Philos Trans R Soc Lond B Biol Sci.

[11] Jones EG (1998). A new view of specific and nonspecific thalamocortical connections.. Adv Neurol.

[12] Guillery RW (1995). Anatomical evidence concerning the role of the thalamus in corticocortical communication: a brief review.. J Anat.

[13] Rouiller EM, Welker E (2000). A comparative analysis of the morphology of corticothalamic projections in mammals.. Brain Res Bull.

[14] Reichova I, Sherman SM (2004). Somatosensory corticothalamic projections: distinguishing drivers from modulators.. J Neurophysiol.

[15] Sherman SM, Guillery RW (1998). On the actions that one nerve cell can have on another: distinguishing “drivers” from “modulators”.. Proc Natl Acad Sci U S A.

[16] Eaton SA, Salt TE (1996). Role of N-methyl-D-aspartate and metabotropic glutamate receptors in corticothalamic excitatory postsynaptic potentials in vivo.. Neuroscience.

[17] Li J, Guido W, Bickford ME (2003). Two distinct types of corticothalamic EPSPs and their contribution to short-term synaptic plasticity.. J Neurophysiol.

[18] Jones EG, Hendry SHC (1989). Differential calcium binding protein immunoreactivity distinguishes classes of relay neurons in monkey thalamic nuclei.. Eur J Neurosci.

[19] Jones EG (1998). Viewpoint: the core and matrix of thalamic organization.. Neuroscience.

[20] Rockland KS (1996). Two types of corticopulvinar terminations: round (type 2) and elongate (type1).. J Comp Neurol.

[21] Rockland KS, Andresen J, Cowie RJ, Robinson DL (1999). Single axon analysis of pulvinocortical connections to several visual areas in the macaque.. J Comp Neurol.

[22] Casagrande VA, Guillery RW, Sherman SM (2005). Cortical function: A view from the thalamus.. Prog Brain Res.

[23] Abbott LF, Chance FS (2005). Drivers and modulators from push-pull and balanced synaptic input.. Prog Brain Res.

[24] Barbas H, Henion TH, Dermon CR (1991). Diverse thalamic projections to the prefrontal cortex in the rhesus monkey.. J Comp Neurol.

[25] McFarland NR, Haber SN (2002). Thalamic relay nuclei of the basal ganglia form both reciprocal and nonreciprocal cortical connections, linking multiple frontal cortical areas.. J Neurosci.

[26] Xiao D, Barbas H (2004). Circuits through prefrontal cortex, basal ganglia, and ventral anterior nucleus map pathways beyond motor control.. Thalamus & Related Systems.

[27] Zikopoulos B, Barbas H (2006). Prefrontal projections to the thalamic reticular nucleus form a unique circuit for attentional mechanisms.. J Neurosci.

[28] Germuska M, Saha S, Fiala J, Barbas H (2006). Synaptic distinction of laminar specific prefrontal-temporal pathways in primates.. Cereb Cortex.

[29] Medalla M, Lera P, Feinberg M, Barbas H (2007). Specificity in inhibitory systems associated with prefrontal pathways to temporal cortex in primates.. Cereb Cortex.

[30] Negyessy L, Goldman-Rakic PS (2005). Morphometric characterization of synapses in the primate prefrontal cortex formed by afferents from the mediodorsal thalamic nucleus.. Exp Brain Res.

[31] Schwartz ML, Dekker JJ, Goldman-Rakic PS (1991). Dual mode of corticothalamic synaptic termination in the mediodorsal nucleus of the rhesus monkey.. J Comp Neurol.

[32] Pieribone VA, Shupliakov O, Brodin L, Hilfiker-Rothenfluh S, Czernik AJ (1995). Distinct pools of synaptic vesicles in neurotransmitter release.. Nature.

[33] Schweizer FE, Ryan TA (2006). The synaptic vesicle: cycle of exocytosis and endocytosis.. Curr Opin Neurobiol.

[34] Becherer U, Rettig J (2006). Vesicle pools, docking, priming, and release.. Cell Tissue Res.

[35] Rollenhagen A, Lubke JH (2006). The morphology of excitatory central synapses: from structure to function.. Cell Tissue Res.

[36] Gabbott PL, Bacon SJ (1996). Local circuit neurons in the medial prefrontal cortex (areas 24a,b,c, 25 and 32) in the monkey: II. Quantitative areal and laminar distributions.. J Comp Neurol.

[37] Dombrowski SM, Hilgetag CC, Barbas H (2001). Quantitative architecture distinguishes prefrontal cortical systems in the rhesus monkey.. Cereb Cortex.

[38] Vidnyanszky Z, Gorcs TJ, Negyessy L, Borostyankio Z, Knopfel T (1996). Immunocytochemical visualization of the mGluR1a metabotropic glutamate receptor at synapses of corticothalamic terminals originating from area 17 of the rat.. Eur J Neurosci.

[39] Ilinsky IA, Kultas-Ilinsky K (1990). Fine structure of the magnocellular subdivision of the ventral anterior thalamic nucleus (VAmc) of Macaca mulatta: I. Cell types and synaptology.. J Comp Neurol.

[40] Wurtz RH, Sommer MA, Cavanaugh J (2005). Drivers from the deep: the contribution of collicular input to thalamocortical processing.. Prog Brain Res.

[41] Guillery RW (2005). Anatomical pathways that link perception and action.. Cortical Function: A View from the Thalamus.

[42] Guillery RW (2003). Branching thalamic afferents link action and perception.. Journal of Neurophysiology.

[43] Guillery RW, Sherman SM (2002). The thalamus as a monitor of motor outputs.. Philosophical Transactions of the Royal Society of London Series B-Biological Sciences.

[44] Kao CQ, Coulter DA (1997). Physiology and pharmacology of corticothalamic stimulation-evoked responses in rat somatosensory thalamic neurons in vitro.. J Neurophysiol.

[45] Golshani P, Warren RA, Jones EG (1998). Progression of change in NMDA, non-NMDA, and metabotropic glutamate receptor function at the developing corticothalamic synapse.. Journal of Neurophysiology.

[46] Turner JP, Salt TE (1998). Characterization of sensory and corticothalamic excitatory inputs to rat thalamocortical neurones in vitro.. J Physiol.

[47] Ojima H (1994). Terminal morphology and distribution of corticothalamic fibers originating from layers 5 and 6 of cat primary auditory cortex.. Cereb Cortex.

[48] Rouiller EM, Simm GM, Villa AE, deRibaupierre Y, deRibaupierre F (1991). Auditory corticocortical interconnections in the cat: evidence for parallel and hierarchical arrangement of the auditory cortical areas.. Exp Brain Res.

[49] Pierce JP, Mendell LM (1993). Quantitative ultrastructure of Ia boutons in the ventral horn: scaling and positional relationships.. J Neurosci.

[50] Pierce JP, Lewin GR (1994). An ultrastructural size principle.. Neuroscience.

[51] Shepherd GM, Harris KM (1998). Three-dimensional structure and composition of CA3–>CA1 axons in rat hippocampal slices: implications for presynaptic connectivity and compartmentalization.. J Neurosci.

[52] Rosenmund C, Stevens CF (1996). Definition of the readily releasable pool of vesicles at hippocampal synapses.. Neuron.

[53] Murthy VN, Sejnowski TJ, Stevens CF (1997). Heterogeneous release properties of visualized individual hippocampal synapses.. Neuron.

[54] Thomson AM (2000). Molecular frequency filters at central synapses.. Prog Neurobiol.

[55] Wang S, Eisenback MA, Bickford ME (2002). Relative distribution of synapses in the pulvinar nucleus of the cat: implications regarding the “driver/modulator” theory of thalamic function.. J Comp Neurol.

[56] Montero VM (1991). A quantitative study of synaptic contacts on interneurons and relay cells of the cat lateral geniculate nucleus.. Exp Brain Res.

[57] Madarasz M, Tombol T, Hajdu F, Somogyi G (1981). Some comparative quantitative data on the different (relay and associative) thalamic nuclei in the cat. A quantitative EM study.. Anat Embryol (Berl).

[58] Liu XB, Honda CN, Jones EG (1995). Distribution of four types of synapse on physiologically identified relay neurons in the ventral posterior thalamic nucleus of the cat.. J Comp Neurol.

[59] Ramcharan EJ, Gnadt JW, Sherman SM (2005). Higher-order thalamic relays burst more than first-order relays.. Proc Natl Acad Sci U S A.

[60] Van Horn SC, Erisir A, Sherman SM (2000). Relative distribution of synapses in the A-laminae of the lateral geniculate nucleus of the cat.. J Comp Neurol.

[61] Lund JS, Lund RD, Hendrickson AE, Hunt AB, Fuchs AF (1976). The origin of efferent pathways from the primary visual cortex, area 17, of the macaque monkey as shown by retrograde transport of horseradish peroxidase.. J Comp Neurol.

[62] Abramson BP, Chalupa LM (1985). The laminar distribution of cortical connections with the tecto- and cortico-recipient zones in the cat's lateral posterior nucleus.. Neuroscience.

[63] Melchitzky DS, Sesack SR, Lewis DA (1999). Parvalbumin-immunoreactive axon terminals in macaque monkey and human prefrontal cortex: laminar, regional, and target specificity of type I and type II synapses.. J Comp Neurol.

[64] Shipp S (2003). The functional logic of cortico-pulvinar connections.. Philos Trans R Soc Lond B Biol Sci.

[65] Lee CC, Sherman SM (2006). The thalamocortical synapse: driver properties from higher order nuclei..

[66] Somogyi P, Tamas G, Lujan R, Buhl EH (1998). Salient features of synaptic organisation in the cerebral cortex.. Brain Res Brain Res Rev.

[67] Peters A, Payne BR (1993). Numerical relationships between geniculocortical afferents and pyramidal cell modules in cat primary visual cortex.. Cereb Cortex.

[68] Kubota Y, Hatada S, Kondo S, Karube F, Kawaguchi Y (2007). Neocortical inhibitory terminals innervate dendritic spines targeted by thalamocortical afferents.. J Neurosci.

[69] Volk DW, Lewis DA (2003). Effects of a mediodorsal thalamus lesion on prefrontal inhibitory circuitry: implications for schizophrenia.. Biol Psychiatry.

[70] Cipolloni PB, Keller A (1989). Thalamocortical synapses with identified neurons in monkey primary auditory cortex: a combined golgi/EM and GABA/peptide immunocytochemistry study.. Brain Res.

[71] Kwegyir-Afful EE, Bruno RM, Simons DJ, Keller A (2005). The role of thalamic inputs in surround receptive fields of barrel neurons.. J Neurosci.

[72] Lewis DA, Cruz DA, Melchitzky DS, Pierri JN (2001). Lamina-specific deficits in parvalbumin-immunoreactive varicosities in the prefrontal cortex of subjects with schizophrenia: evidence for fewer projections from the thalamus.. Am J Psychiatry.

[73] Hoover JE, Strick PL (1993). Multiple output channels in the basal ganglia.. Science.

[74] Devinsky O, Morrell MJ, Vogt BA (1995). Contributions of anterior cingulate cortex to behaviour.. Brain.

[75] Sabatini U, Boulanouar K, Fabre N, Martin F, Carel C (2000). Cortical motor reorganization in akinetic patients with Parkinson's disease: a functional MRI study.. Brain.

[76] Conn PJ, Battaglia G, Marino MJ, Nicoletti F (2005). Metabotropic glutamate receptors in the basal ganglia motor circuit.. Nat Rev Neurosci.

[77] Llinas RR, Ribary U, Jeanmonod D, Kronberg E, Mitra PP (1999). Thalamocortical dysrhythmia: A neurological and neuropsychiatric syndrome characterized by magnetoencephalography.. Proc Natl Acad Sci U S A.

[78] Garcia-Cabezas MA, Rico B, Sanchez-Gonzalez MA, Cavada C (2007). Distribution of the dopamine innervation in the macaque and human thalamus.. Neuroimage.

[79] Lewis DA, Foote SL, Goldstein M, Morrison JH (1988). The dopaminergic innervation of monkey prefrontal cortex: A tyrosine hydroxylase immunohistochemical study.. Brain Res.

[80] Goldman-Rakic PS, Lidow MS, Smiley JF, Williams MS (1992). The anatomy of dopamine in monkey and human prefrontal cortex.. Journal of Neural Transmission - Supplementum.

[81] Mizuno A, Villalobos ME, Davies MM, Dahl BC, Muller RA (2006). Partially enhanced thalamocortical functional connectivity in autism.. Brain Res.

[82] Herbert MR, Ziegler DA, Makris N, Filipek PA, Kemper TL (2004). Localization of white matter volume increase in autism and developmental language disorder.. Ann Neurol.

[83] Courchesne E, Pierce K (2005). Why the frontal cortex in autism might be talking only to itself: local over-connectivity but long-distance disconnection.. Curr Opin Neurobiol.

[84] Larkum ME, Senn W, Luscher HR (2004). Top-down dendritic input increases the gain of layer 5 pyramidal neurons.. Cereb Cortex.

[85] Zikopoulos B, Barbas H (2007). Circuits for multisensory integration and attentional modulation through the prefrontal cortex and the thalamic reticular nucleus in primates.. Rev Neurosci.

[86] Rubenstein JL, Merzenich MM (2003). Model of autism: increased ratio of excitation/inhibition in key neural systems.. Genes Brain Behav.

[87] Jones EG (2001). The thalamic matrix and thalamocortical synchrony.. Trends Neurosci.

[88] Xiao D, Barbas H (2002). Pathways for emotions and memory II. afferent input to the anterior thalamic nuclei from prefrontal, temporal, hypothalamic areas and the basal ganglia in the rhesus monkey.. Thalamus and Related Systems.

[89] Barbas H, Medalla M, Alade O, Suski J, Zikopoulos B (2005). Relationship of prefrontal connections to inhibitory systems in superior temporal areas in the rhesus monkey.. Cereb Cortex.

[90] Richmond FJR, Gladdy R, Creasy JL, Ditamura S, Smits E (1994). Efficacy of seven retrograde tracers, compared in multiple-labelling studies of feline motoneurones.. J Neurosci Meth.

[91] Veenman CL, Reiner A, Honig MG (1992). Biotinylated dextran amine as an anterograde tracer for single- and double-labeling studies.. J Neurosci Methods.

[92] Reiner A, Veenman CL, Medina L, Jiao Y, Del Mar N (2000). Pathway tracing using biotinylated dextran amines.. J Neurosci Methods.

[93] Jones EG, Tighilet B, Tran BV, Huntsman MM (1998). Nucleus- and cell-specific expression of NMDA and non-NMDA receptor subunits in monkey thalamus.. J Comp Neurol.

[94] McCormick DA, von Krosigk M (1992). Corticothalamic activation modulates thalamic firing through glutamate “metabotropic” receptors.. Proc Natl Acad Sci U S A.

[95] Godwin DW, Van Horn SC, Erisir A, Sesma M, Romano C (1996). Ultrastructural localization suggests that retinal and cortical inputs access different metabotrophic glutamate receptors in the lateral geniculate nucleus.. J Neurosci.

[96] Olszewski J (1952). The Thalamus of the *Macaca mulatta*. An Atlas for Use with the Stereotaxic Instrument..

[97] Barbas H, Pandya DN (1989). Architecture and intrinsic connections of the prefrontal cortex in the rhesus monkey.. J Comp Neurol.

[98] Walker AE (1940). A cytoarchitectural study of the prefrontal area of the macaque monkey.. J Comp Neurol.

[99] Howard CV, Reed MG (1998). Unbiased Stereology, Three-dimensional Measurement in Microscopy..

[100] Gundersen HJ (1986). Stereology of arbitrary particles. A review of unbiased number and size estimators and the presentation of some new ones, in memory of William R. Thompson.. J Microsc.

[101] West MJ, Slomianka L, Gundersen HJG (1991). Unbiased stereological estimation of the total number of neurons in the subdivisions of the rat hippocampus using the optical fractionator.. Anat Rec.

[102] Fiala JC (2005). Reconstruct: a free editor for serial section microscopy.. J Microsc.

[103] Hashikawa T, Molinari M, Rausell E, Jones EG (1995). Patchy and laminar terminations of medial geniculate axons in monkey auditory cortex.. J Comp Neurol.

[104] Peters A, Palay SL, Webster HD (1991). The fine structure of the nervous system. Neurons and their supporting cells..

[105] Barbas H, Saha S, Rempel-Clower N, Ghashghaei T (2003). Serial pathways from primate prefrontal cortex to autonomic areas may influence emotional expression.. BMC Neurosci.

