# Supplementary figures and images for: Parallel Driving and Modulatory Pathways Link the Prefrontal Cortex and Thalamus
Source: PLoS One. 2007 Sep 5;2(9):e848. doi: 10.1371/journal.pone.0000848 (PMC1952177; doi:10.1371/journal.pone.0000848)

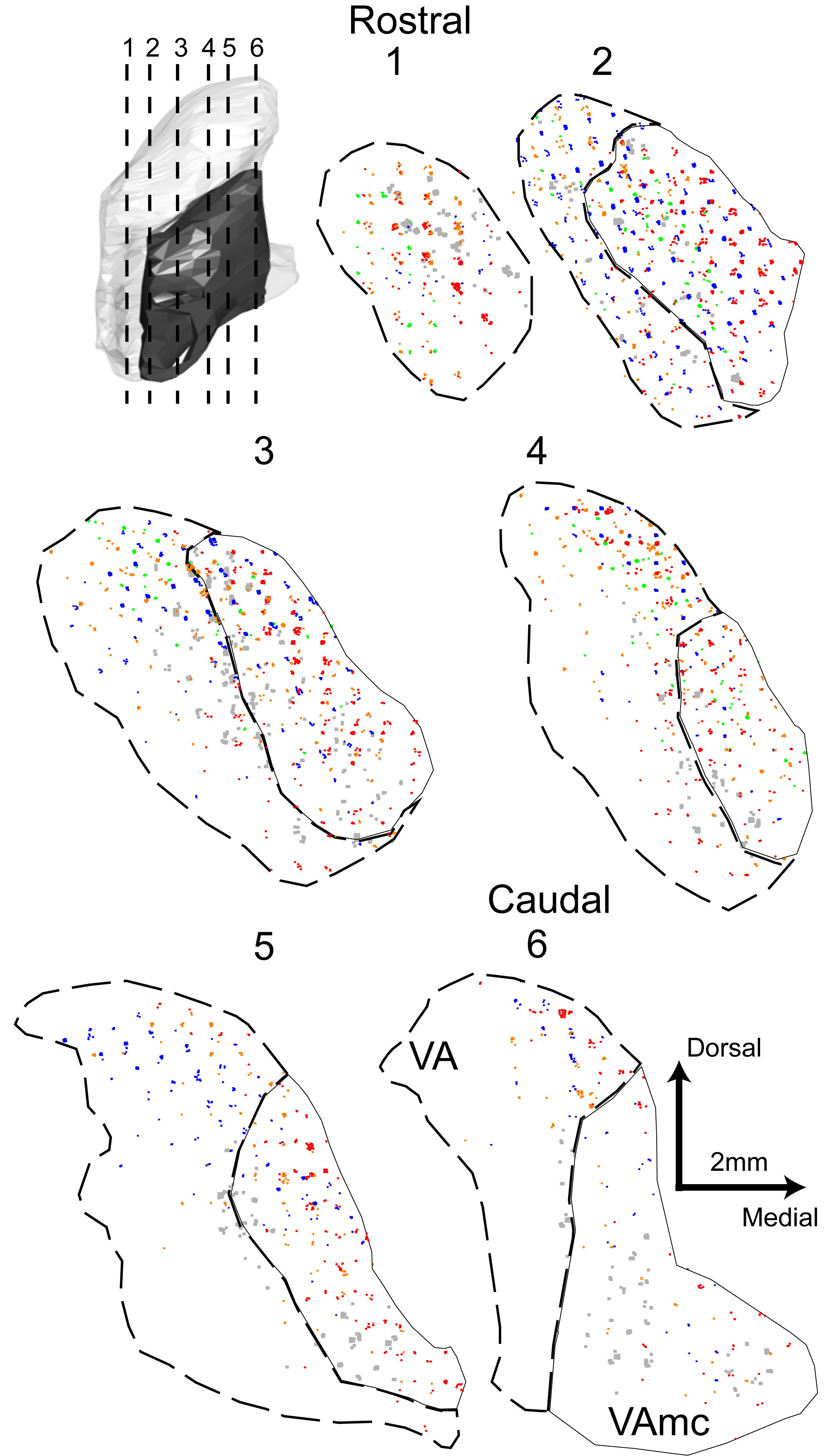

Supplement: Figure S1 — Prefrontal axonal terminations in the ventral anterior nuclei. Two-dimensional quantitative projection patterns in six representative coronal sections covering the whole rostrocaudal extent of the ventral anterior nucleus (dotted black outline) and its magnocellular part (black outline), shown in 3D-reconstruction (top, left). Boutons from prefrontal axons are represented by colored dots as follows: red, area 32; blue, area 9; green, area 10; orange, area 46; grey, orbital area 13. Each dot represents approximately 50 boutons. (0.62 MB TIF) [file pone.0000848.s001.tif]
