# Supplementary material for: Parallel Driving and Modulatory Pathways Link the Prefrontal Cortex and Thalamus
Source: PLoS One. 2007 Sep 5;2(9):e848. doi: 10.1371/journal.pone.0000848 (PMC1952177; doi:10.1371/journal.pone.0000848)
Supplement: Table S1 — (0.03 MB DOC) [file pone.0000848.s002.doc]

| **Table S1:** Injection sites and neural tracers used in 10 cases analyzed | | | |
| --- | --- | --- | --- |
|  | | | |
| **Case Name** | **Injection Site** | **Brain Hemisphere** | **Tracer** |
| AY | 32 | Left | BDA |
| BA | 10 | Right | BDA |
| BC | 10 | Left | BDA |
|  | 13 | Right | FE |
| BD | VA | Left | FE |
| BE | VA | Left | FR |
|  | VA | Left | FB |
| BF | 10 | Left | BDA |
| BG | 9 | Left | BDA |
|  | 32 | Right | BDA |
|  | VA | Right | FE |
|  | VA | Right | FR |
| BH | Dorsal 46 | Left | BDA |
|  | 9 | Right | FR |
|  | 9 | Right | FB |
|  | 13 | Right | LY |
| BI | 32 | Right | BDA |
|  | Dorsal 46 | Left | FR |
|  | 13 | Left | LY |
|  | 10 | Left | FB |
| BJ | 9 | Right | BDA |
|  | 9 | Left | FE |

# 
