# Supplementary material for: Parallel Driving and Modulatory Pathways Link the Prefrontal Cortex and Thalamus
Source: PLoS One. 2007 Sep 5;2(9):e848. doi: 10.1371/journal.pone.0000848 (PMC1952177; doi:10.1371/journal.pone.0000848)
Supplement: Text S1 — (0.03 MB DOC) [file pone.0000848.s003.doc]

## Text S1: Quantitative distribution of prefrontal terminations in the ventral anterior nucleus

Data on prefrontal terminations in the ventral anterior nucleus were obtained after injection of anterograde tracers in prefrontal cortices shown on the medial (Fig. 12A), lateral (Fig. 12B) and basal (Fig. 12C) surfaces of the brain. We mapped the distribution of labeled terminals from cortical axons in the ventral anterior nucleus in one series of sections (1/10 of the total nucleus) and estimated stereologically the axonal bouton density.

Labeled prefrontal axons entered the ventral anterior nucleus rostrally and laterally from the adjacent internal capsule passing through the inhibitory thalamic reticular nucleus. A majority of prefrontal cortico-thalamic terminals (≥ 70%) were found in largely overlapping sites of the rostral half of both nuclei (Fig. S1). Clusters of labeled projection neurons were embedded in areas of axonal terminations, with the latter always occupying larger zones. Overall, the magnocellular part of the ventral anterior nucleus had a higher density of labeled terminals, ranging from 36,492 – 333,566 boutons/mm^3^ (mean: 163,280 ± 37,463), compared to the ventral anterior nucleus in the same cases, which ranged from 4,294 – 277,813 boutons/mm^3^ (mean: 100,180 ± 33,053). The distribution of prefrontal terminals was similar to a previous study [25]. Briefly, axons from area 46 terminated in distinct patches mainly in the medial parts of the ventral anterior nucleus and the lateral parts of its magnocellular part. Terminations from area 10 showed a similar distribution in the rostral region of the magnocellular part but were significantly less dense. In comparison with lateral prefrontal areas, the connections of medial areas 9 and 32 were more robust and encompassed larger regions of the medial ventral anterior nucleus and lateral magnocellular ventral anterior nucleus. Posteriorly, terminations occupied the dorsomedial parts of the ventral anterior nucleus and medial regions of its magnocellular part. Labeled axonal terminations from orbital area 13 were prominent in the ventromedial part of the ventral anterior nucleus, and the ventrolateral part of its magnocellular part, and there was significant overlap with projections from medial prefrontal areas.
