# Supplementary material for: Parallel Driving and Modulatory Pathways Link the Prefrontal Cortex and Thalamus
Source: PLoS One. 2007 Sep 5;2(9):e848. doi: 10.1371/journal.pone.0000848 (PMC1952177; doi:10.1371/journal.pone.0000848)
Supplement: Text S2 — (0.04 MB DOC) [file pone.0000848.s004.doc]

## Text S2: Postsynaptic targets of prefrontal axonal boutons onto thalamic neurons

Some studies in other brain regions and systems have indicated that large boutons synapse primarily on proximal dendrites and cell bodies, whereas small boutons synapse on distal dendrites [88–90]. However, in this prefrontal cortico-thalamic pathway both large and small boutons synapsed on similar dendrites with an average diameter of 1.51 μm ± 0.69, a size that includes both proximal and distal dendrites, consistent with a previous study in this system [32]. We confirmed this finding after intracellular injections of fluorescent dyes in PV+ and CB+ thalamic projection neurons, which had been labeled retrogradely from prefrontal cortical injections. Intracellular injections of projection neurons filled the dendritic tree more fully and made it possible to reconstruct the neurons in three dimensions and measure the diameters of their proximal and distal dendrites. Proximal dendrites included the primary and secondary branches, which had diameters ranging from 1 to 6.5 μm. All other parts of the dendritic tree were distal, and their diameter ranged from 0.2 to 2.5 μm. Therefore, the sizes of proximal and distal dendrites overlapped between 1 to 2.5 μm. There were no significant differences in the diameters of dendritic trees of CB+ and PV+ neurons (Fig. 8).
